# Inhibition of a Novel CLK1-THRAP3-PPARγ Axis Improves Insulin Sensitivity

**DOI:** 10.3389/fphys.2021.699578

**Published:** 2021-08-30

**Authors:** Zhenguo Wang, Xiaojing Gao, Qingrun Li, Hongwen Zhu, Xiangjie Zhao, Minerva Garcia-Barrio, Jifeng Zhang, Yanhong Guo, Y. Eugene Chen, Rong Zeng, Jia-Rui Wu, Lin Chang

**Affiliations:** ^1^Department of Internal Medicine, Cardiovascular Center, University of Michigan Medical Center, Ann Arbor, MI, United States; ^2^CAS Key Laboratory of Systems Biology, Hangzhou Institute for Advanced Study, University of Chinese Academy of Sciences, Chinese Academy of Sciences, Hangzhou, China; ^3^Key Laboratory of Systems Biology, CAS Center for Excellence in Molecular Cell Science, Institute of Biochemistry and Cell Biology, University of Chinese Academy of Sciences, Chinese Academy of Sciences, Shanghai, China; ^4^School of Life Sciences and Technology, Shanghai Tech University, Shanghai, China

**Keywords:** insulin sensitivity, browning, CLK1, THRAP3, PPARγ

## Abstract

Increasing energy expenditure by promoting “browning” in adipose tissues is a promising strategy to prevent obesity and associated diabetes. To uncover potential targets of cold exposure, which induces energy expenditure, we performed phosphoproteomics profiling in brown adipose tissue of mice housed in mild cold environment at 16°C. We identified CDC2-like kinase 1 (CLK1) as one of the kinases that were significantly downregulated by mild cold exposure. In addition, genetic knockout of CLK1 or chemical inhibition in mice ameliorated diet-induced obesity and insulin resistance at 22°C. Through proteomics, we uncovered thyroid hormone receptor-associated protein 3 (THRAP3) as an interacting partner of CLK1, further confirmed by co-immunoprecipitation assays. We further demonstrated that CLK1 phosphorylates THRAP3 at Ser243, which is required for its regulatory interaction with phosphorylated peroxisome proliferator-activated receptor gamma (PPARγ), resulting in impaired adipose tissue browning and insulin sensitivity. These data suggest that CLK1 plays a critical role in controlling energy expenditure through the CLK1-THRAP3-PPARγ axis.

## Introduction

Obesity features a massive expansion of white adipose tissue (WAT) in the visceral and subcutaneous regions due to excess energy storage in adipocytes (Choe et al., 2016). Obese people are at much higher risk for diabetes and cardiovascular complications (Akil and Ahmad, 2011). Therefore, preserving homeostatic energy storage within healthy levels in adipose tissues could be a major strategy to prevent obesity and related diabetes and cardiovascular complications (Sun et al., 2011). The finding that functional brown adipose tissue (BAT) exists in adult humans (Nedergaard et al., 2007, 2010) and its characteristics of high energy expenditure (Fruhbeck et al., 2009; Cypess and Kahn, 2010; Haas et al., 2012; Heeren and Munzberg, 2013) underlie BAT’s emergence as a potential intrinsic target for prevention of obesity, diabetes and related cardiovascular diseases (CVDs). It is well-known that cold environmental temperature is the most efficient strategy to activate thermogenesis and energy expenditure in BAT (Nedergaard et al., 2007), concurrent with a complex physiological response marked by the increase in food intake, oxygen consumption and heat generation (Robidoux et al., 2004). WAT undergoes a “browning” process in response to cold temperature (Fisher et al., 2012; Ohno et al., 2012; Lin et al., 2017), suggesting that cold stimulus mobilizes energy metabolism in both WAT and BAT. Stimulating “browning” in WAT could improve metabolic health by reducing the intrinsic adverse effects of WAT, and conferring beneficial effects on hyperlipidaemia and insulin resistance (Bartelt and Heeren, 2014). Due to the small amount of active BAT in adult humans, stimulating WAT “browning” is an attractive alternative strategy to promote metabolic health through increasing energy expenditure in the “browning” fat. Although environmental cold exposure strongly stimulates release of norepinephrine to acutely activate thermogenesis, environmental temperature is an important determinant of cardiac sympathetic and parasympathetic outflow, which in turn has a major impact on the cardiovascular system (Silvani et al., 2016). There is a significant negative correlation between the environmental temperature and the blood pressure and heart rate in older patients (Postolache et al., 1993). Consistently, seasonal morbidity and mortality due to CVDs is significantly increased in both the northern and southern hemispheres during the winter rather than in the summer (Manou-Stathopoulou et al., 2015). Therefore, it would be unrealistic to induce BAT activation by cold stimulation in humans. Some regulators of transcription such as PR/SET Domain 16 (PRDM16, a transcriptional coregulator that controls the development of brown adipocytes in BAT), and peroxisome proliferator-activated receptor gamma (PPARγ), hormones such as irisin and fibroblast growth factor 21 (FGF21) and chemical compounds such as thiazolidinediones (TZDs) were reported to induce WAT browning, increase energy expenditure, and protect mice from diet-induced obesity (Seale et al., 2011; Boström et al., 2012; Fisher et al., 2012; Ohno et al., 2012; Cohen et al., 2014). To date, none of those is applied clinically to induce WAT browning and prevent obesity and associated diseases.

Phosphorylation and dephosphorylation of proteins (including enzymes and receptors) is one of the crucial mechanisms to promote energy expenditure in response to cold stimuli (Ardito et al., 2017). The balanced action of protein kinases (for phosphorylation) and phosphoprotein phosphatases (for dephosphorylation) determines the overall protein phosphorylation state. Due to the importance of protein phosphorylation in biological process, tremendous efforts have been made to identify the various functions of protein kinase signal transduction pathways (Manning et al., 2002; Roskoski, 2015). Since cold exposure is a robust environmental stress, it will undoubtedly induce a series of phosphorylation and dephosphorylation events in thermogenic adipose tissues. Our goal is to explore novel protein kinase signaling pathways able to mimic moderate cold stimuli in adipose tissues with the objective of identifying potential targets for pharmacologic intervention. Therefore, we performed phosphoproteomics analysis in BAT from mice housed at 16°C. We identified that the kinase activity of CDC2-like kinase 1 (CLK1) in BAT is significantly reduced upon chronic mild cold exposure. It is well-established that CLK1, a dual-specificity tyrosine and serine/threonine protein kinase, plays critical roles in alternative splicing through phosphorylation of SR proteins. However, CLK1 function, regulation, and targets in a metabolic context are relatively unknown. In this study, we investigated the roles of CLK1 in metabolism under the hypothesis that inhibition of CLK1 may promote adipose tissue browning. We show here that inhibition of CLK1 improved insulin sensitivity by preventing phosphorylation of THRAP3 (thyroid hormone receptor-associated protein 3, a transcriptional cofactor) at Ser243. THRAP3 is an RNA-binding protein which regulates circadian clock-dependent alternative splicing of pre-mRNAs (Lande-Diner et al., 2013; Marcheva et al., 2020), androgen-independent prostate cancer cell growth (Ino et al., 2016), and adipocyte differentiation (Katano-Toki et al., 2013). It was reported that THRAP3 can directly interact with PPARγ when the latter is phosphorylated on Ser273 in adipocytes (Choi et al., 2014). Our data uncovered that phosphorylation on Ser243 of THRAP3 by CLK1 increased preferential docking of THRAP3 to PPARγ phosphorylated on Ser273 in adipocytes, which inhibits PPAR γ activity (Choi et al., 2014). Inhibition of CLK1 reduced THRAP3 phosphorylation and herein prevented docking to PPARγ, resulting in decreased PPARγ phosphorylation on Ser273, thus enhancing PPARγ activity to promote adipocyte browning, insulin signaling and glucose metabolism. These data strongly suggest that CLK1–THRAP3–PPARγ complexes could be a potent therapeutic target for obesity and associated type 2 diabetes.

## Materials and Methods

Detailed methods are described in the supplemental document.

### Animal Study

C57BL/6J (stock number 000664) or diet-induced obese (DIO) male mice (stock number 380050) were purchased from The Jackson Laboratory. We generated *Clk1* knockout mice (CLK1 KO) in the C57BL/6J background using CRISPR/Cas9 technology, as described in detail in the supplemental document. All mice used for the studies were male and were housed in ventilated cages at either 22 or 16°C with 12h/12h light/dark cycle, with the dark phase starting at 6 pm. Mice had *ad libitum* access to standard chow diet (CD, D12450J, Research Diets, 20% proteins, 10% fat, 70% carbohydrate) or high-fat diet (HFD, D12492, Research Diets, 20% proteins, 60% fat, 20% carbohydrate) and water. The animal numbers used for each experiment are indicated in the corresponding figure legends. The study protocol was approved by the Institutional Animal Care and Use Committee of the University of Michigan.

### Energy Expenditure Assay in Mice

Oxygen consumption (VO_2_), carbon dioxide production (VCO_2_), spontaneous motor activity and food intake were measured using the Comprehensive Laboratory Monitoring System (CLAMS, Columbus Instruments), an integrated open-circuit calorimeter equipped with an optical beam activity monitoring device as we described before (Xiong et al., 2017). Total energy expenditure was calculated based on the values of VO_2_, VCO_2_, and the protein breakdown (Riachi et al., 2004).

### Glucose and Insulin Tolerance Assay

For the glucose tolerance test (GTT), D-glucose (2 mg/g of body weight) was orally gavaged to 5-h fasted mice and glucose levels were monitored at 0, 30, 60, and 120 min subsequently using a Glucometer Elite (Bayer, Japan). For the Insulin-tolerance test (ITT), the experiments were performed on mice following a 5-h fast. Animals were injected intraperitoneally with 0.5 U/kg body weight of human insulin (Humulin, Eli Lilly Co., Indianapolis, IN, United States). Tail-blood samples were taken at 0, 30, 60, and 90 min after injection for measurement of blood glucose levels.

### Mass Spectrometry for Phosphoproteomics

Male C57BL/6J wild-type mice (8-week-old) were fed a standard chow diet and housed at 16 or 22°C for 8 weeks. The BAT was collected and lysed in SDT buffer (4%SDS, 0.1 M DTT, 100 mM Tris-HCl, pH7.6) and homogenized using Precellys24 Homogenizer (Bertin Technologies). Phosphorylated peptides were separated by EASY-nLC 1000 C18 liquid chromatography (Thermo Fisher Scientific) and analyzed by Orbitrap Fusion (Thermo Fisher Scientific). For phosphoproteome analysis, raw mass spectrometry data were processed using the MaxQuant software version 1.5.2.8 and peak lists were analyzed against the mouse Uniprot database.

### Statistical Analysis

The data were evaluated with two-tailed, unpaired Student’s *t*-test or compared by One-way ANOVA with Dunnett’s multiple comparisons test or Two-way ANOVA with Sidak’s multiple comparisons test and were expressed as mean ± SEM or SD. A value of *p* < 0.05 was considered statistically significant. Although presented in the same graph for convenience, the independent expression of each given gene across the different tissues listed in the X axis was analyzed through pair-wise comparison by two-tailed, unpaired Student’s *t*-test.

## Results

### Phosphoproteomics Profiling of BAT Identifies Downregulation of CLK Kinase Activity in Response to Mild Cold Stimulation

**FIGURE 1:**
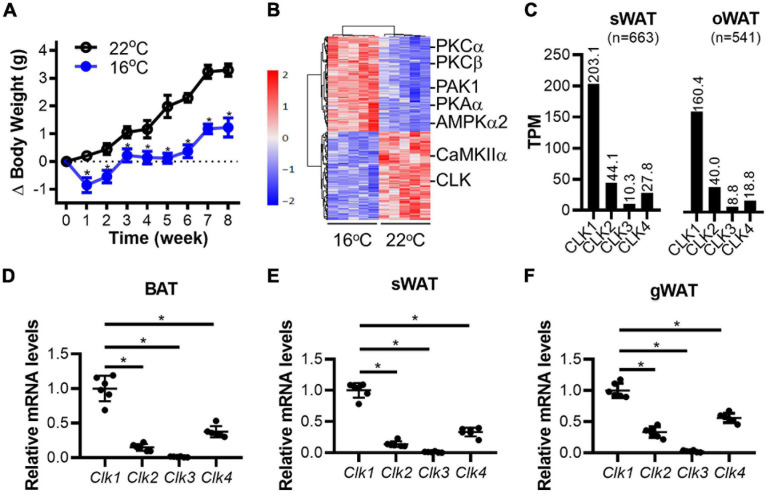
Downregulation of CLK in brown adipose tissue in mice housed in a mild cold environment. **(A)** The body weight change of 8-week-old male mice fed a standard chow diet for 8 weeks in either 22 or 16°C environmental chambers. *n* = 6/group, data shown as mean ± SEM, *p < 0.05 vs 22°C. (Two-way ANOVA with Sidak’s multiple comparisons test). **(B)** At the end of the experiment described in panel **(A)**, the brown adipose tissue (BAT) in the interscapular region was collected and subjected to phosphoproteomics by LC-MS/MS. The hierarchical clustering and heatmap shows the sites differentially phosphorylated in response to mild-cold as determined in BAT. The color scale represents the relative abundance of sites. Further analysis identified CLK as one of the down-regulated family of kinases in interscapular BAT in mice housed at 16°C. *n* = 5/group. **(C)** Expression of the CLK family members in human subcutaneous (sWAT) and omental adipose tissues (oWAT). The data was adapted from the GTEx Portal (https://www.gtexportal.org/home/gene/CLK1). **(D–F)** The mRNA levels of the *Clk* family members (relative to 18S rRNA) in adipose tissues from interscapular (BAT), subcutaneous (sWAT) and gonadal (gWAT) regions of 8-week-old C57BL/6J mice on standard chow diet and housed at 22°C. The relative *Clk1* mRNA levels in adipose tissues were set as 1, *n* = 6/group, data shown as mean ± SD, *p < 0.05 vs *Clk1*. One-way ANOVA with Sidak’s multiple comparisons test.

Cold-induced thermogenesis is associated with the coordinate mobilization of glucose, lipid, and protein metabolism, which involves activation/inactivation of the corresponding enzymes in the corresponding metabolic pathways in multiple organs. As shown in Figure 1A, the body weight gain in mice fed a standard chow diet was significantly blunted when housed at 16°C (mild-cold environment) in comparison to mice housed at 22°C (standard room temperature). To identify changes in the signaling pathways in adipose tissues of mice housed at 16°C, a multiplex TMT based quantitative phosphoproteome strategy was applied as depicted in Supplementary Figure 1. After exposure of 8-week-old mice to 16 or 22°C in environmental chambers for 8 weeks, the BAT in the interscapular region was collected and analyzed by LC-MS/MS. Next, the differentially phosphorylated sites identified in proteins from BAT were analyzed to determine the functional kinases responsible for the cold-induced phosphorylation profiles. We identified 9,306 phosphorylation sites on 3,120 phosphorylated proteins, of which 6,810 phosphorylation sites on 2,775 phosphorylated proteins could be accurately localized without missing values (class I sites, localization probability >0.75, score diff >5, Supplementary Table 1) (Rigbolt et al., 2011). To compare phosphorylation changes in BAT in response to mild-cold exposure, hierarchical clustering was applied based on 662 sites to show 343 up-regulated and 319 down-regulated phosphorylation sites (adjusted *p* value < 0.05 of *t*-test, fold change >1.5). Those sites were further analyzed to predict the corresponding upstream kinases using NetworKIN 3.0 (Horn et al., 2014) and explore the overrepresented kinases using a Hypergeometric test. We identified both activated kinases, including PKCα, PKCβ, PAK1, PKAα, AMPKα2, and inhibited kinases, including CLK and CaMKIIα, upon mild-cold exposure (Figure 1B). Of relevance, PKA and AMPK signaling were reported to be activated in BAT under cold stimulation (Mulligan et al., 2007; Harms and Seale, 2013), consistent with our finding here. However, although the CLK represent one of the strongest inhibited kinase family upon mild-cold exposure, the contribution of their signaling pathway to thermogenesis and metabolism remains unknown. There are four genes in the CLK family in human (*CLK1*, *CLK2*, *CLK3*, and *CLK4*). Because of their high sequence conservation, we could not identify *a priori* which member in the CLK family was inhibited in BAT upon mild-cold exposure. Western blotting showed that the expression levels of the CLK family are higher in adipose tissues than those in other metabolic organs such as liver, heart, brain and skeletal muscle. CLK1 was mainly expressed in adipose tissues. Interestingly, expression of CLK1 in BAT, a thermogenic tissue, is lower than that in subcutaneous (sWAT) and gonadal WAT (gWAT). While CLK2 was widely expressed in different organs with higher abundance in adipose tissues. CLK3 has been reported predominantly expressed in the testis (Nayler et al., 1997). Our results show that adipose tissues and heart express high level of CLK3 as well. Beside adipose tissues, CLK4 is also highly expressed in liver, brain and testis, in which CLK1 is barely expressed (Supplementary Figure 2). By searching the gene expression profile in the GTEx Portal,^1^ we found that CLK1 was the most enriched CLK family member in both human subcutaneous and omental adipose tissue (Figure 1C). We further demonstrated that the mRNA levels of *Clk1* were significantly higher than those of other *Clk* family members in both BAT and WAT of mice (Figures 1D–F). These data suggest that CLK1 is the predominant kinase in the CLK family in adipose tissues and might contribute to thermogenesis and metabolism in response to cold exposure.

### CLK1 Is Highly Expressed in Brown-Like Adipose Tissues and Is Negatively Associated With Thermogenesis

**FIGURE 2:**
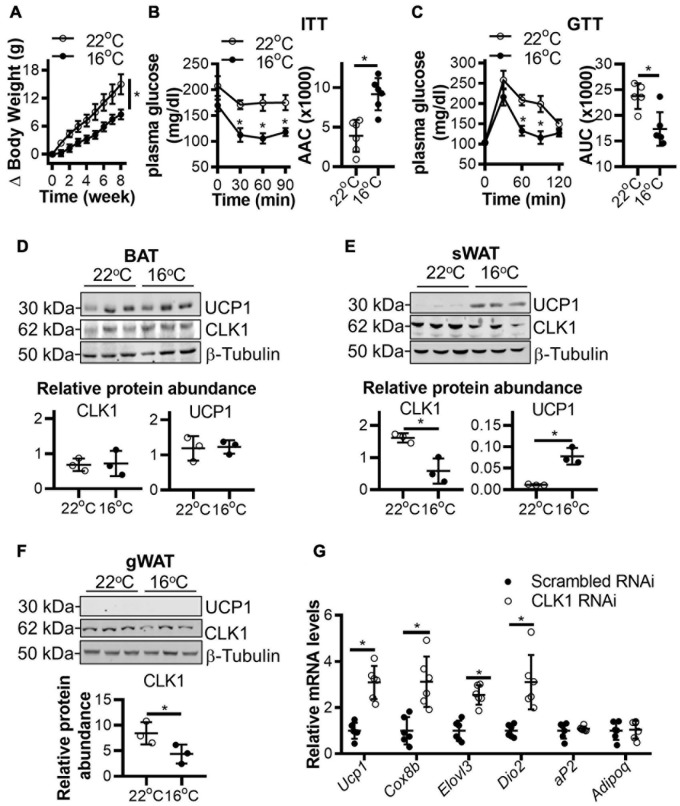
CLK1 downregulation in adipose tissues is associated with insulin sensitivity in mice housed in a mild cold environment. **(A)** The body weight change of 8-week-old male mice being fed a high-fat diet (HFD) for 8 weeks while housed in either 22 or 16°C environmental chambers. *n* = 6/group, data shown as mean ± SEM, *p < 0.05 vs 22°C. (Two-way ANOVA with Sidak’s multiple comparisons test). **(B)** The insulin tolerance test (ITT) and **(C)** glucose tolerance test (GTT) in mice from panel **(A)** at the end of the 8-weeks of HFD feeding. Histograms show the corresponding areas above the curves (AAC) of ITT, and areas under the curves (AUC) of GTT. *n* = 6/group, data shown as mean ± SEM, *p < 0.05 vs 22°C. (Two-way ANOVA with Sidak’s multiple comparisons test). **(D–F)** The protein abundance of CLK1 and UCP1 in BAT **(D)**, sWAT **(E)**, and gWAT **(F)** in mice from panel **(A)** at the end point of the 8-week HFD feeding. The lower panels are the quantitative data of the corresponding Western blots shown in the upper panels. *n* = 3/group, data shown as mean ± SD, *p < 0.05 vs 22°C. (Unpaired Student’s *t* test). **(G)** The mRNA levels (relative to 18S rRNA) of browning markers in immortalized mouse brown adipocytes, originally isolated from mice on chow diet, and subjected to *Clk1* knockdown or siRNA control (10 nM) on day 5 of differentiation and treatment at differentiation day 7 with 10 mM CL316,243 for 24 h. *n* = 3, data shown as mean ± SD, *p < 0.05 vs scrambled RNAi control. (Unpaired Student’s *t* test).

To further validate the association of CLK1 expression in adipose tissues with obesity, C57BL/6J mice were housed at 22 or 16°C for 8 weeks and given a HFD. As shown in Figure 2A, the body weight gain was significantly reduced in mice housed at 16°C when compared to those housed at 22°C. Meanwhile, insulin and glucose tolerance were improved in the mice housed at 16°C (Figures 2B,C). Mild-cold exposure did not significantly alter uncoupling protein 1 (UCP1) abundance in BAT, but UCP1 was significantly increased in sWAT from obese mice. CLK1 protein abundance was comparable in BAT in obese mice housed at either 22 or 16°C. However, CLK1 was reduced in sWAT and gonadal WAT (gWAT) of mice housed at 16°C when compared to those at 22°C (Figures 2D–F), suggesting that CLK1 in obese sWAT and gWAT was negatively regulated in cold exposure. Even though CLK1 protein abundance was not significantly changed in BAT of obese mice, knockdown of *Clk1* in isolated mouse brown adipocytes increased mRNA levels of brown adipocyte markers such as *Ucp1*, *Cox8b*, *Elovl3*, and *Dio2* upon treatment with CL316,243, a β3-adrenergic receptor agonist (Figure 2G).

**FIGURE 3:**
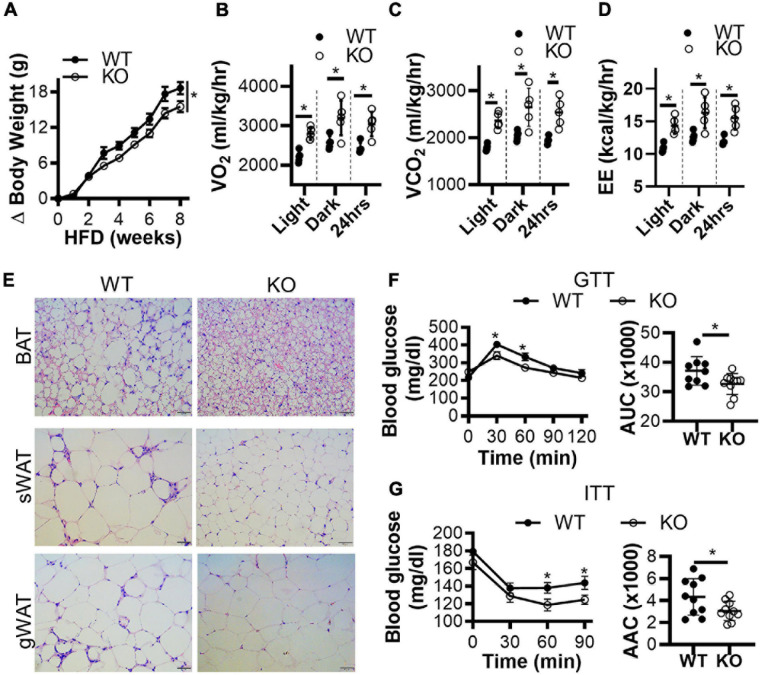
Genetic knockout of CLK1 improves insulin sensitivity in mice. **(A)** The body weight gain of male CLK1 KO mice (KO) and wild-type littermate controls (WT) fed a high-fat diet (HFD) for 8 weeks, starting at 8 weeks of age and housed at regular room temperature at 22°C. *n* = 10 mice/group. Data shown as mean ± SEM. *p < 0.05 vs WT. (One-way ANOVA with Sidak’s multiple comparisons test). **(B–D)** The whole-body energy expenditure of CLK1 KO mice in panel **(A)** were measured at the end of the 8 weeks of HFD feeding. The environmental temperature in the metabolic chambers was set at 22°C. The metabolic index was monitored for 24 h, and the results were averaged during the light-on period, and during the light-off period, respectively. *n* = 5 mice/group. Data shown as mean ± SD. *p < 0.05 vs WT. (Unpaired Student’s *t* test). **(E)** H&E staining of BAT, sWAT, and gWAT at the end of the 8 weeks of HFD feeding in panel **(A)**, scale bar, 100 μm. **(F)** The glucose tolerance test (GTT) and **(G)** the insulin tolerance test (ITT); the histograms show the corresponding areas under the curves (AUC) for GTT, and areas above the curves (AAC) for ITT. *n* = 10 mice/group. Data shown as mean ± SEM. (One-way ANOVA with Sidak’s multiple comparisons test).

**FIGURE 4:**
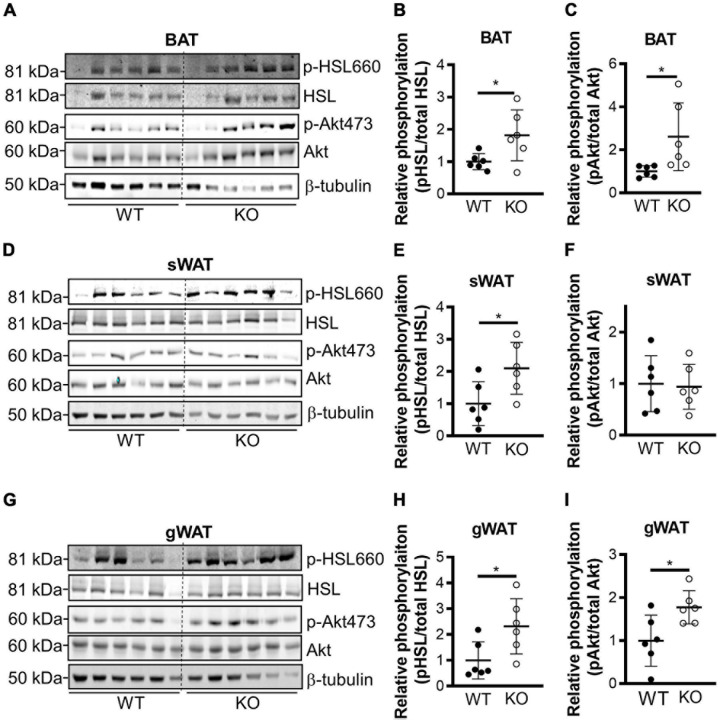
Genetic knockout of CLK1 increases phosphorylation of HSL and Akt. Western blot analysis of the levels of phosphorylation of HSL on Ser660 and Akt on Ser473, with the corresponding densitometric quantification (as the ratio phospho/total protein) in the indicated adipose tissues from WT and KO mice at the end of 8 weeks’ HFD feeding as in Figure 3A. BAT **(A–C)**, sWAT **(D–F)**, and gWAT **(G–I)**. *n* = 6 mice/group. Data shown as mean ± SD, *p < 0.05. (Unpaired Student’s *t* test). The mean of WT was set as 1 for comparison.

Next, we generated conventional *Clk1* knockout mice (CLK1 KO) (Supplementary Figures 3A,B) to investigate whether CLK1 contributes to metabolism *in vivo*. We further confirmed that *Clk1* in BAT and sWAT was negatively associated with expression of thermogenic genes at 22°C (Supplementary Figures 3C,D). The adipocyte browning markers were significantly increased in sWAT from CLK1 KO mice, and the mRNA abundance of genes associated with thermogenesis such as *Elovl3*, *Dio2*, and *Pgc1a* was increased in BAT from CLK1 KO mice when compared to those from littermate wild-type control mice. The body weight gain in CLK1 KO mice was significantly less than in littermate wild-type controls after HFD feeding for 8 weeks (Figure 3A). The oxygen consumption, carbon dioxide production and energy expenditure were significantly increased in the CLK1 KO mice after the 8 weeks’ HFD feeding (Figures 3B–D). Since these metabolic indexes were comparable between the wild-type and CLK1 KO mice, either as absolute values per mouse or normalized per lean body mass (Supplementary Figures 4A,B), the increased metabolism in the CLK1 KO mice might be due to reduced fat mass. The histological analysis of adipose tissues indicated that *Clk1* knockout prevented BAT whitening and WAT hypertrophy in HFD, as evidenced by reduced lipid droplet sizes in both BAT and WAT (Figure 3E). Additionally, CLK1 KO mice showed improved glucose and insulin tolerance compared with wild-type control mice (Figures 3F,G). In agreement with the lean phenotype in the CLK1 KO mice, the phosphorylation of hormone sensitive lipase (HSL) at Ser660 which promotes its lipolytic activity (Adebonojo et al., 1982), was significantly increased in all types of adipose tissue when compared to those in wild-type mice (Figures 4A,B,D,E,G,H). The phosphorylation of protein kinase B (Akt) at Ser473, which is considered as a surrogate of insulin activity (Krook et al., 1997), was increased in BAT and gWAT in the CLK1 KO mice as well (Figures 4A,C,D,F,G,I). These data suggest that *Clk1* knockout promotes thermogenesis and energy expenditure, resulting in a lean phenotype and underscoring the potential benefit of pharmacologic inhibition of CLK1 for the prevention of obesity and associated diabetes.

### Identification of THRAP3 as a New Substrate of CLK1

**FIGURE 5:**
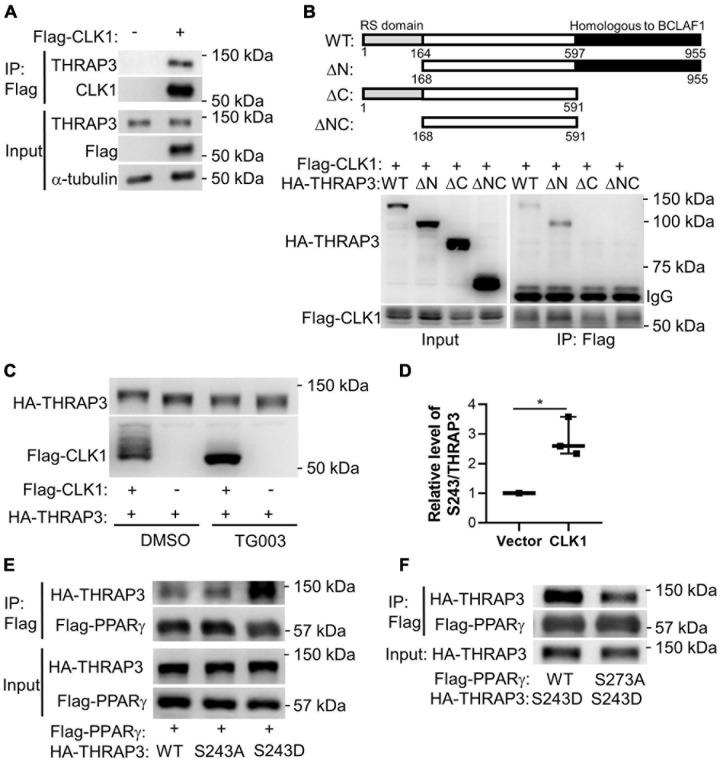
Functional complexes of CLK1-THRAP3-PPARγ. **(A)** Flag-tagged CLK1 transfected into HEK293T cells was pulled down with an anti-Flag antibody after 48 h. THRAP3 co-immunoprecipitation (Co-IP) was verified by western blot. **(B)** Schematic illustration of full length and truncated mutants of THRAP3 **(top)**. Flag-tagged CLK1 and HA-tagged THRAP3 full length (WT) or truncated mutants (ΔN, ΔC, or ΔNC) were co-transfected into HEK293T cells and subjected to subsequent immunoprecipitation using anti-Flag antibody. Anti-HA antibody was used to detect THRAP3 bound to CLK1 in the Western blots **(bottom)**. **(C)** Co-transfection of Flag-tagged CLK1 and HA-tagged full length THRAP3 into HEK293T cells followed by treatment with the CLK1 inhibitor TG003 (10 μM) or vehicle control DMSO for 6 h. The cell lysates were collected and subjected to Western blotting. The mobility shift in both THRAP3 and CLK1 in the presence of the CLK1 inhibitor is indicative of increased THRAP3 phosphorylation and CLK1 autophosphorylation in the presence of overexpressed CLK1 without the inhibitor (heavier bands, lane 1). **(D)** Co-transfection of HA-tagged THRAP3 and Flag-tagged CLK1, or empty vector as control, into HEK293T cells for 24 h was followed by immunoprecipitation with anti-HA monoclonal antibody-conjugated agarose beads. The relative phosphorylation of THRAP3 at Ser243 in the immunoprecipitants was measured by Mass Spectrometry and was normalized relative to the vector control HEK293T cells set as 1. *n* = 5. *p < 0.05 vs Vector. (Unpaired Student’s *t* test). **(E)** HA-tagged phosphorylation-deficient (S243A) or phosphorylation-mimic (S243D) forms of THRAP3 were co-transfected with Flag-PPARγ into HEK293T cells. Following Flag-mediated Co-IP, their interactions were analyzed by western blotting. **(F)** HA-THRAP3 S243D mutants were co-expressed with the WT or S273A forms of Flag-PPARγ in HEK293T cells. The interactions were analyzed by Co-IP assays using anti-HA antibody followed by western blotting with anti-Flag antibody.

We further applied a proteomics approach to uncover binding partners of CLK1. For this purpose, HA-tagged CLK1 was overexpressed in HEK293T cells and cell lysates were immunoprecipitated with anti-HA monoclonal antibody-conjugated agarose beads. The resulting co-immunoprecipitants were then subjected to LC-MS/MS analysis. We identified 88 proteins that were efficiently pulled down by HA-tagged CLK1 (>1.5 fold than control empty vector), and THRAP3 was the most abundant protein among them (∼43 times higher than control, Supplementary Table 2). We further confirmed that CLK1 physically interacts with THRAP3 using co-immunoprecipitation (Co-IP) followed by western blot analysis (Figure 5A). As previously described (Vohhodina et al., 2017), THRAP3 has an SR-rich (serine/arginine) domain in the N-terminal region and a domain homologous to Bcl-2-associated transcription factor 1 (BCLAF1) in the C-terminus. To further determine the THRAP3 domain necessary for binding with CLK1, full-length and truncated versions of THRAP3 lacking the N-terminal, C-terminal or N- and C-terminal regions, respectively, were co-expressed with Flag-tagged CLK1 in HEK293T cells and subjected to Co-IP assays using anti-Flag monoclonal antibody-conjugated agarose beads followed by western blot analysis. The results indicated that the C-terminal domain of THRAP3 is required for the interaction with CLK1 (Figure 5B). When CLK1 and THRAP3 were co-expressed in HEK293T cells, the THRAP3 band shifted to a slightly heavier molecular weight by SDS-PAGE, while this mobility shift was abolished upon treatment with TG003, a potent CLK family inhibitor (Muraki et al., 2004), suggesting that CLK1 could phosphorylate THRAP3 (Figure 5C). The inhibitor also increased CLK1 mobility, indicative of autophosphorylation. Additionally, we also uncovered that phosphorylation on multiple sites in THRAP3, including S243, S253, and S379, were significantly reduced in BAT upon 16°C cold stimulation (Supplementary Table 1), which is consistent with the downregulation of the CLK1 kinase activity in response to mild cold stimulation. To further establish that CLK1 phosphorylates THRAP3, we co-expressed HA-tagged THRAP3 and Flag-tagged CLK1 in HEK293T cells. The immunoprecipitated THRAP3 was then analyzed for phosphorylation by mass spectrometry. As shown in Figure 5D, compared to control (empty vector), overexpression of CLK1 significantly increased THRAP3 phosphorylation at S243. Collectively, these data indicate that CLK1 binds to the C-terminal domain of THRAP3 and phosphorylates it at S243.

### CLK1-Phosphorylation on S243 in THRAP3 Promotes Its PPARγ-Binding Activity

**FIGURE 6:**
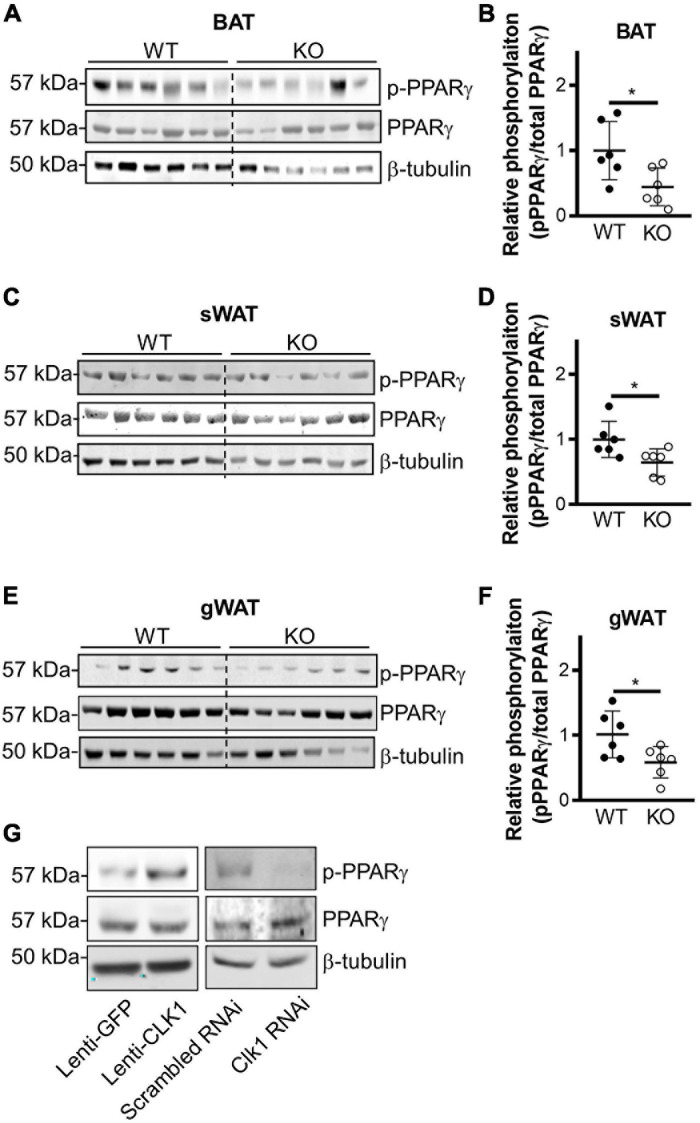
CLK1 deficiency reduces phosphorylation of PPARγ on Ser273. **(A–F)** Male CLK1 KO (KO) or wildtype (WT) mice were fed a HFD for 8 weeks, starting at 8 weeks of age. Western blot (left panel) and quantitative densitometry data (right panel) showing phosphorylation of PPARγ in BAT **(A,B)**, sWAT **(C,D)**, and gWAT **(E,F)** in WT and KO mice at the end point of the 8-week’s HFD feeding. Data shown as mean ± SD, *p < 0.05. (Unpaired Student’s *t* test). **(G)** Western blot showing phosphorylation of PPARγ in immortalized mouse brown adipocytes with CLK1 overexpression (10 MOI of lentivirus, Lenti-GFP as control) or knockdown (10 nM of siRNA, scrambled siRNA as control).

It was reported that THRAP3 can directly interact with PPARγ when the latter is phosphorylated at S273 (Choi et al., 2014). We confirmed that wild-type THRAP3 interacts with PPARγ, and further demonstrated that THRAP3 S243D (mimicking constitutive phosphorylation) increases binding to PPARγ, while THRAP3 S243A (phosphorylation-deficient mutant) fails to increase the pulldown (Figure 5E). Furthermore, mutation of PPARγ S273 (S273A) significantly blocked PPARγ and THRAP3 interaction (Figure 5F). These data indicate that phosphorylation of S243 in THRAP3 and S273 in PPARγ are critical for their interaction. Indeed, the phosphorylation of S273 in PPARγ is reduced in all types of adipose tissues in the CLK1 KO mice (Figures 6A–F). Consistently, overexpression of CLK1 increased, while knockdown of CLK1 reduced phosphorylation of PPARγ at S273 in brown adipocytes (Figure 6G). These data imply that reduced phosphorylation of THRAP3 at 243 and of PPARγ at 273 might contribute to improved insulin sensitivity in the CLK1 KO mice.

### Chemical Inhibition of CLK Improves Insulin Sensitivity in Obese Mice

**FIGURE 7:**
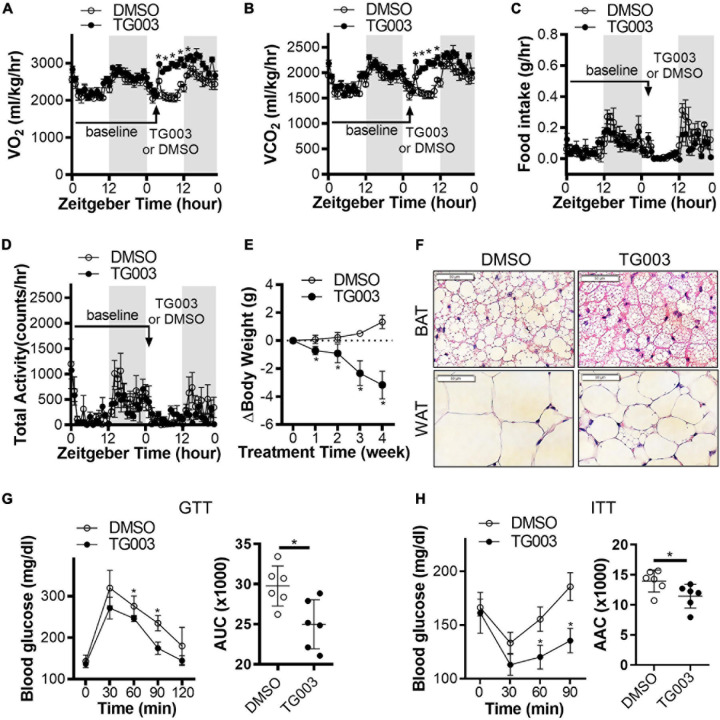
Inhibition of CLK improves insulin sensitivity in obese mice. **(A–D)** Diet induced obesity (DIO) in male C57BL/6J mice was induced by high-fat diet (HFD) for 24 weeks starting at 6 weeks of age, at 22°C. Energy expenditure in obese mice was measured at baseline for 24 h after 24 weeks of HFD feeding. After subcutaneous injection one bolus of CLK inhibitor TG003 (50 μg/kg) or vehicle control DMSO (black arrow), the energy expenditure was measured for another 24 h. Gray area indicates the light-off period. *n* = 5 mice/group. Data shown as mean ± SEM. *p < 0.05 vs DMSO. (One-way ANOVA with Sidak’s multiple comparisons test). **(E)** After the energy expenditure measurements in panels **(A–D)**, the DIO mice continued the HFD feeding while being treated with TG003 (50 μg/kg) or DMSO (subcutaneous injection, once a day) for an additional 4 weeks. The body weight change of mice was calculated by subtracting the body weight on the first day of TG003 or DMSO treatment from that of each week of TG003 or DMSO treatment. *n* = 6 mice/group. Data shown as mean ± SEM. *p < 0.05 vs DMSO. (One-way ANOVA with Sidak’s multiple comparisons test). **(F)** H&E staining of the interscapular brown adipose tissue (BAT) and subcutaneous white adipose tissue (WAT), scale bar, 50 μm. **(G,H)** The oral glucose tolerance test **(G)** and insulin tolerance test **(H)**, at the end point of HFD feeding and TG003 or DMSO treatment as described in panel **(E)**. Histograms show the corresponding areas under the curves (AUC) of GTT and areas above the curves (AAC) of ITT. *n* = 6 mice/group. Data shown as mean ± SD. *p < 0.05 vs DMSO. [One-way ANOVA with Sidak’s multiple comparisons test for the curves in panels **(G,H)** and unpaired Student’s *t* test for histograms in panels **(G,H)**].

**FIGURE 8:**
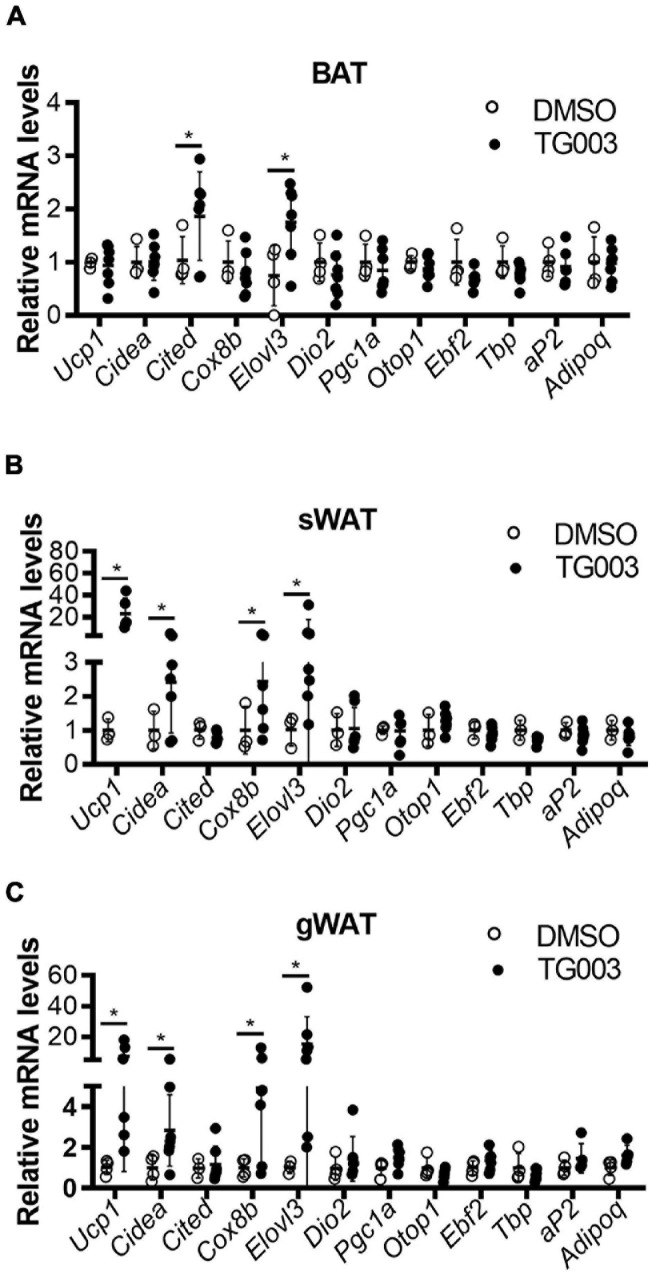
TG003 treatment increases expression of markers of browning in adipose tissues. **(A–C)** mRNA levels of browning marker genes in BAT **(A)**, sWAT **(B)**, and gWAT **(C)** at the end point of HFD feeding combined with TG003 or DMSO treatment, as described in Figure 7E. *n* = 6 mice/group. Data shown as mean ± SD. *p < 0.05 vs DMSO. (Unpaired Student’s *t* test for each gene).

To evaluate the potential translational application of these findings, we investigated the effects of CLK1 inhibition on whole body metabolism and insulin sensitivity. Wild-type C57BL/6J mice were fed an HFD for 24 weeks to induce obesity and diabetes while housed at 22°C. At 24 weeks, the mice were treated with TG003 (50 mg/kg). We found that after subcutaneous injection of a single dose of TG003 there was significantly increased oxygen consumption and carbon dioxide production without affecting the food intake and total activity (Figures 7A–D and Supplementary Figures 5A,B). Next, we continued treatment of the same obese mice with TG003 for an additional 4 weeks while keeping them on HFD. As shown in Figure 7E, the body weight of obese mice significantly declined during the TG003 treatment. Consistently, the sizes of lipid droplets in adipocytes in both BAT and gWAT were significantly smaller in the TG003-treated mice compared to the DMSO-treated mice (Figure 7F). Glucose and insulin tolerance tests showed that TG003 treatment of the obese mice significantly improved insulin sensitivity (Figures 7G,H), in association with the body weight decrease. Additionally, TG003 treatment significantly increased the expression of the browning markers *Ucp1*, *Cidea*, *Cox8b*, and *Elovl3* in sWAT and gWAT, and of *Cited* and *Elovl3* in BAT (Figures 8A–C). These data indicate that CLK1 inhibition induced WAT browning and could improve insulin sensitivity while reducing obesity in a WT obese mice model.

## Discussion

Cold exposure is the most potent stimulus to activate BAT or induce “browning” of WAT. The thermogenic function of BAT or the “browning” of fat is tightly controlled by norepinephrine released from the sympathetic nervous system (Cannon and Nedergaard, 2004). Although environmental cold exposure strongly stimulates the release of norepinephrine to acutely activate thermogenesis, environmental temperature is an important determinant of cardiac sympathetic and parasympathetic outflow, which in turn has a major impact on the cardiovascular system (Silvani et al., 2016). The heart rate and blood pressure of mice were reduced when the housing temperature was raised from 20 to 30°C, and increased when lowered from 30 to 20°C (Swoap et al., 2008). During mild/moderate cold exposure (12–17°C), the body core temperature was significantly decreased and the blood pressure was increased in aging healthy subjects (Inoue et al., 1992) and in hypertensive patients as well (Hess et al., 2009). In fact, there is a significant negative correlation between the environmental temperature and the blood pressure and heart rate in older patients (Postolache et al., 1993). Consistently, seasonal morbidity and mortality due to CVDs is significantly increased in both the northern and southern hemispheres during the winter rather than in the summer (Manou-Stathopoulou et al., 2015). Even though we previously demonstrated that a mild cold environment at 16°C significantly induced adipose tissue browning and prevented atherosclerosis development in mice (Chang et al., 2012; Xiong et al., 2017), it is unrealistic and could be somewhat dangerous for humans to be exposed to cold environments. In this study, we defined inhibition of CLK1 as a potential strategy to induce WAT browning and prevent obesity and associated diabetes as an alternative to cold exposure.

The CLK family of evolutionarily conserved dual-specificity kinases consists of four isoforms (CLK1-4) found in most tissues and cell types. Despite high homology among the CLKs, each member may have a distinct biology in different tissues. CLK1 was identified as a kinase that regulates pre-mRNA splicing by catalyzing the phosphorylation of Serine and arginine-rich (SR) RNA binding proteins (Duncan et al., 1997; Dufresne et al., 1998). Hyper-phosphorylated SR proteins bind to pre-mRNA and stabilize the interactions of spliceosome components, promoting spliceosome assembly (Mermoud et al., 1994). CLK1 also directly phosphorylates and activates the mitogen-activated protein kinase signaling cascade, including ERK1/ERK2 and PTP-1B (Moeslein et al., 1999), and the splicing factor SPF45, a non-SR protein (Liu et al., 2013). CLK1 also activates the kinetochore protein kinase KKT2 *via* phosphorylation at the S508 residue, which is crucial for kinetochore localization and function during cell division (Saldivia et al., 2021). Additionally, CLK1 functions as a component of a wider signaling network. Akt serine/threonine kinase 2 (Akt2), belonging to a subfamily of serine/threonine kinases containing SH2-like (Src homology 2-like) domains, can phosphorylate CLK1 and promote CLK1-mediated phosphorylation of SR proteins (Jiang et al., 2009). Those CLK1-associated signaling pathways regulate cell migration and invasion are linked to tumor development and progression. Notably, SM08502, a CLK1 inhibitor, recently entered clinical trials for the treatment of advanced solid tumors (Tam et al., 2020). Additionally, pharmacological inhibition of CLK1 has been investigated pre-clinically for the treatment of Duchenne muscular dystrophy (Sako et al., 2017) and Alzheimer’s disease (Jain et al., 2014; Hedou et al., 2016; Murar et al., 2017). Beyond these diseases, it was reported that lower temperature causes CLK1 activation *in vitro* in cultured HEK293 cells (Haltenhof et al., 2020). It is unknown if CLK1 activity is altered when the body temperature changes in mammals. By screening the cold-induced phosphoproteome, we found that CLK activity was significantly reduced in BAT from mice housed in a mild-cold environment.

The protein abundance of CLK1 upon mild cold exposure was markedly reduced in sWAT and gWAT, which may account for the reduced kinase activity of CLK1. Meanwhile, in BAT, CLK1 protein abundance was unchanged while the kinase activity was significantly reduced upon mild cold exposure, raising the possibility that post-translational modifications of CLK1 play a predominant role in the regulation of CLK1 kinase activity in BAT, which will be the object of future studies. As a dual-specificity protein kinase, CLK1 has been reported to auto-phosphorylate on tyrosine residues (Menegay et al., 2000). Similarly, although the BAT is the major thermogenic organ in rodents, the expression levels of UCP1 and other thermogenic genes in BAT were less responsive to cold stimuli when compared to those in beige adipose tissues. The mechanisms underlying this phenomenon remain unknown.

We further showed that CLK1 deficient mice were resistant to HFD-induced obesity under regular room temperature conditions. Unlike the phenotype of the *Clk1* knockout mice reported here, overexpression of CLK2 in the mediobasal thalamus can partially reverse the HFD-induced obese phenotype in mice (Quaresma et al., 2017), and mice lacking *Clk2* in adipose tissue exhibited exacerbated obesity (Hatting et al., 2017). The underlying mechanisms of the reversed phenotype on obesity between CLK1 and CLK2 are unknown and will be further explored in future studies. This phenomenon was also observed in a study of HIV-1 replication. CLK1 had opposite effects to those of CLK2 on viral replication. CLK1 enhanced Gag production while CLK2 inhibited the virus, while CLK3 and CLK4 had no significant effect on viral replication (Wong et al., 2011). Phosphorylated cAMP-response element binding protein (CREB) is a transcriptional activator of UCP1. CLK2 decreases CREB dephosphorylation in a protein phosphatase 2-dependent manner. Deletion of *Clk2* in adipose tissues reduced energy expenditure in mice as well as UCP1 abundance in brown adipocytes due to enhanced CREB dephosphorylation (Hatting et al., 2017). Contrasting the lower energy expenditure in CLK2 knockout mice, the CLK1 deficient mice showed enhanced energy expenditure per body weight. However, the energy expenditure normalized by lean body mass or without normalization was comparable between wildtype and *Clk1* knockout mice, suggesting that less fat mass or maintenance of functional fat contributed to the enhanced energy expenditure in the CLK1 deficient mice. Furthermore, we found that Akt phosphorylation levels were strongly elevated in BAT of CLK1 KO mice, presumably involving a feedback mechanism. But how Akt regulates CLK1 kinase activity and the potential feedback of CLK1 on Akt kinase activity will require further investigation. It was reported that inhibition of CLK1 blocked adipocyte differentiation *in vitro*. 3T3-L1 cells carrying mutations of putative Akt phosphorylation sites in CLK1 failed to form lipid droplets during differentiation (Li et al., 2013). Our study showed that the lipid droplets of adipocytes were smaller in WAT and BAT of CLK1 KO mice than those in WT mice, which might be also due to the reduced differentiation capability of CLK1 deficient preadipocytes.

We additionally found that the lack or inhibition of CLK1 improves glucose tolerance and preserves insulin sensitivity upon HFD feeding. This phenotype might be solely due to reduced fat mass since it is well-known that dysfunctional fat accumulation causes insulin resistance.

We further uncovered a novel pathway defined by a CLK1-THRAP3-PPARγ axis which might impair insulin sensitivity under obese conditions. THRAP3 is a transcriptional cofactor containing an SR domain and has been identified as a component of the spliceosome which is required for pre-mRNA splicing and it activates splicing *in vivo* (Lee et al., 2010). Loss of THRAP3 results in sensitivity to DNA damaging agents, genomic instability, and defective DNA repair, which are in themselves promising targets for chemotherapy of cancers (Vohhodina et al., 2017). Additionally, THRAP3 acts as a cofactor of sex-determining region Y-box 9 (SOX9) and negatively regulates the transcriptional activity of the SOX9 complex during chondrogenesis (Sono et al., 2018). However, the roles of THRAP3 in energy metabolism are largely unknown. THRAP3 binds the helicase motifs in helicase with zinc finger 2 (HELZ2). HELZ2 and THRAP3 synergistically augmented transcriptional activation mediated by PPARγ in differentiated 3T3-L1 cells (Katano-Toki et al., 2013), suggesting that THRAP3 and HELZ2 interaction contributes to adipocyte differentiation through activation of PPARγ-mediated gene expression. THRAP3 has also been shown to directly interact with PPARγ to control diabetic gene programming (Choi et al., 2014). In this study, we showed that CLK1 could phosphorylate THRAP3 at Ser243, and promotes its docking to PPARγ when it is phosphorylated at Ser273 and resulting in inhibition of PPARγ activity (Choi et al., 2014). Consistent with the reduced CLK1 kinase activity in BAT upon mild cold exposure, the phosphorylation levels of THRAP3 at Ser243 were also markedly decreased. Thus, inhibition of CLK1 could block THRAP3 phosphorylation and increase PPARγ activity. Moreover, mild cold exposure altered other phosphorylation sites on THRAP3 in BAT (Supplementary Table 1), implying that THRAP3 may actively participate in the regulation of thermogenesis. Further studies will focus on the interaction of the different phosphorylated forms of THRAP3 with PPARγ and other key transcription factors, and the functional consequences of those interactions for thermogenesis, adipocyte homeostasis and systemic glucose metabolism.

Peroxisome proliferator-activated receptor gamma is one of the most effective targets to improve insulin signaling. PPARγ ligands such as thiazolidinediones were widely used for control of type 2 diabetes in the clinic. Nevertheless, thiazolidinediones showed multiple side effects such as fluid retention, obesity, and congestive heart failure, which led to the withdrawal of rosiglitazone from the market. However, it is still unknown whether those side effects are only PPARγ-dependent or -independent, as well. Post-transcriptional modification of Ser273 on PPARγ is critical for its anti-diabetic roles. Inhibition of phosphorylation of PPARγ on Ser273 could be a key therapeutic mechanism for full and partial agonists or non-agonist drugs targeting PPARγ. It has been established that PPARγ phosphorylation at Ser273 promotes insulin resistance in obese and diabetic mice, and classical PPARγ ligands such as TZDs inhibit Ser273 phosphorylation to improve insulin sensitivity (Choi et al., 2010). Interestingly, inhibition of PPARγ Ser273 phosphorylation by non-agonist ligands was also anti-diabetic (Kamenecka et al., 2010; Choi et al., 2011; Li et al., 2011; Khim et al., 2020). ERK directly phosphorylated Ser273 of PPARγ. Accordingly, inhibition of ERK significantly improved insulin resistance in diabetic mice (Banks et al., 2015). The relationship between ERK and CLK1 is unknown. Whether this underlies the inhibition of PPARγ Ser273 phosphorylation will be addressed in follow up studies. It was suggested that interfering with docking of THRAP3 on PPARγ could be a strategy to screen for compounds for diabetes treatment (Choi et al., 2014). In this study, we found that CLK1 binds THRAP3 and phosphorylates it on Ser243, thus promoting THRAP3 interaction with PPARγ when the latter is phosphorylated on Ser273 (Choi et al., 2014), suggesting that a CLK1-THRAP3-PPARγ axis regulates insulin sensitivity. Additionally, our results indicate that the mRNA levels of adipocyte browning markers are negatively correlated with CLK1 in adipose tissue of obese mice, with *Clk1* knockout significantly increasing adipocyte browning.

In summary, the present study provides evidence that knockout or inhibition of CLK1 will prevent obesity and improve insulin resistance. Our findings support further exploration of pharmacologic inhibition of CLK1 as a potential new treatment for obesity associated diabetes, beyond oncology, Duchenne muscular dystrophy and Alzheimer’s disease.

## Data Availability Statement

The datasets presented in this study can be found in online repositories. The names of the repository/repositories and accession number(s) can be found below: ProteomeXchange Consortium (accession no: PXD027958) via the iProX partner repository (Ma et al., 2019).

## Ethics Statement

The animal study was reviewed and approved by University of Michigan.

## Author Contributions

ZW, XG, QL, HZ, XZ, and LC conducted the experiments. RZ, J-RW, and LC designed the experiments. ZW, MG-B, and LC wrote the manuscript. JZ, MG-B, YG, RZ, and YEC contributed to the data interpretation. All authors contributed to the article and approved the submitted version.

## Conflict of Interest

The authors declare that the research was conducted in the absence of any commercial or financial relationships that could be construed as a potential conflict of interest.

## Publisher’s Note

All claims expressed in this article are solely those of the authors and do not necessarily represent those of their affiliated organizations, or those of the publisher, the editors and the reviewers. Any product that may be evaluated in this article, or claim that may be made by its manufacturer, is not guaranteed or endorsed by the publisher.

## Footnotes

1
https://www.gtexportal.org/home/gene/CLK1

## References

[B1] Adebonojo F. O. Coates P. M. Cortner J. A. (1982). Hormone-sensitive lipase in human adipose tissue, isolated adipocytes, and cultured adipocytes. *Pediatr. Res.* 16 982–988. 10.1203/00006450-198212000-00002 7155675

[B2] Akil L. Ahmad H. A. (2011). Relationships between obesity and cardiovascular diseases in four southern states and Colorado. *J. Health Care Poor Underserved* 22 61–72. 10.1353/hpu.2011.0166 22102306 PMC3250069

[B3] Ardito F. Giuliani M. Perrone D. Troiano G. Lo Muzio L. (2017). The crucial role of protein phosphorylation in cell signaling and its use as targeted therapy (Review). *Int. J. Mol. Med.* 40 271–280. 10.3892/ijmm.2017.3036 28656226 PMC5500920

[B4] Banks A. S. McAllister F. E. Camporez J. P. Zushin P. J. Jurczak M. J. Laznik-Bogoslavski D. (2015). An ERK/Cdk5 axis controls the diabetogenic actions of PPARgamma. *Nature* 517 391–395. 10.1038/nature13887 25409143 PMC4297557

[B5] Bartelt A. Heeren J. (2014). Adipose tissue browning and metabolic health. *Nat. Rev. Endocrinol.* 10 24–36. 10.1038/nrendo.2013.204 24146030

[B6] Boström P. Wu J. Jedrychowski M. P. Korde A. Ye L. Lo J. C. (2012). A PGC1-α-dependent myokine that drives brown-fat-like development of white fat and thermogenesis. *Nature* 481 463–468. 10.1038/nature10777 22237023 PMC3522098

[B7] Cannon B. Nedergaard J. (2004). Brown adipose tissue: function and physiological significance. *Physiol. Rev.* 84 277–359. 10.1152/physrev.00015.2003 14715917

[B8] Chang L. Villacorta L. Li R. Hamblin M. Xu W. Dou C. (2012). Loss of perivascular adipose tissue on peroxisome proliferator-activated receptor-gamma deletion in smooth muscle cells impairs intravascular thermoregulation and enhances atherosclerosis. *Circulation* 126 1067–1078. 10.1161/CIRCULATIONAHA.112.104489 22855570 PMC3493564

[B9] Choe S. S. Huh J. Y. Hwang I. J. Kim J. I. Kim J. B. (2016). Adipose tissue remodeling: its role in energy metabolism and metabolic disorders. *Front. Endocrinol.* 7:30. 10.3389/fendo.2016.00030 27148161 PMC4829583

[B10] Choi J. H. Banks A. S. Estall J. L. Kajimura S. Bostrom P. Laznik D. (2010). Anti-diabetic drugs inhibit obesity-linked phosphorylation of PPARgamma by Cdk5. *Nature* 466 451–456. 10.1038/nature09291 20651683 PMC2987584

[B11] Choi J. H. Banks A. S. Kamenecka T. M. Busby S. A. Chalmers M. J. Kumar N. (2011). Antidiabetic actions of a non-agonist PPARgamma ligand blocking Cdk5-mediated phosphorylation. *Nature* 477 477–481. 10.1038/nature10383 21892191 PMC3179551

[B12] Choi J. H. Choi S. S. Kim E. S. Jedrychowski M. P. Yang Y. R. Jang H. J. (2014). Thrap3 docks on phosphoserine 273 of PPARgamma and controls diabetic gene programming. *Genes Dev.* 28 2361–2369. 10.1101/gad.249367.114 25316675 PMC4215181

[B13] Cohen P. Levy J. D. Zhang Y. Frontini A. Kolodin D. P. Svensson K. J. (2014). Ablation of PRDM16 and beige adipose causes metabolic dysfunction and a subcutaneous to visceral fat switch. *Cell* 156 304–316. 10.1016/j.cell.2013.12.021 24439384 PMC3922400

[B14] Cypess A. M. Kahn C. R. (2010). Brown fat as a therapy for obesity and diabetes. *Curr. Opin. Endocrinol. Diabetes Obes.* 17 143–149. 10.1097/MED.0b013e328337a81f 20160646 PMC3593105

[B15] Dufresne M. Bailey J. A. Dron M. Langin T. (1998). clk1, a serine/threonine protein kinase-encoding gene, is involved in pathogenicity of *Colletotrichum lindemuthianum* on common bean. *Mol. Plant Microbe Interact.* 11 99–108. 10.1094/MPMI.1998.11.2.99 9450334

[B16] Duncan P. I. Stojdl D. F. Marius R. M. Bell J. C. (1997). In vivo regulation of alternative pre-mRNA splicing by the Clk1 protein kinase. *Mol. Cell. Biol.* 17 5996–6001. 10.1128/MCB.17.10.5996 9315658 PMC232448

[B17] Fisher F. M. Kleiner S. Douris N. Fox E. C. Mepani R. J. Verdeguer F. (2012). FGF21 regulates PGC-1alpha and browning of white adipose tissues in adaptive thermogenesis. *Genes Dev.* 26 271–281. 10.1101/gad.177857.111 22302939 PMC3278894

[B18] Fruhbeck G. Becerril S. Sainz N. Garrastachu P. Garcia-Velloso M. J. (2009). BAT: a new target for human obesity? *Trends Pharmacol. Sci.* 30 387–396. 10.1016/j.tips.2009.05.003 19595466

[B19] Haas B. Schlinkert P. Mayer P. Eckstein N. (2012). Targeting adipose tissue. *Diabetol. Metab. Syndr.* 4:43. 10.1186/1758-5996-4-43 23102228 PMC3568051

[B20] Haltenhof T. Kotte A. De Bortoli F. Schiefer S. Meinke S. Emmerichs A. K. (2020). A conserved kinase-based body-temperature sensor globally controls alternative splicing and gene expression. *Mol. Cell* 78 57–69.e4. 10.1016/j.molcel.2020.01.028 32059760

[B21] Harms M. Seale P. (2013). Brown and beige fat: development, function and therapeutic potential. *Nat. Med.* 19 1252–1263. 10.1038/nm.3361 24100998

[B22] Hatting M. Rines A. K. Luo C. Tabata M. Sharabi K. Hall J. A. (2017). Adipose tissue CLK2 promotes energy expenditure during high-fat diet intermittent fasting. *Cell Metab.* 25 428–437. 10.1016/j.cmet.2016.12.007 28089567 PMC5299049

[B23] Hedou D. Godeau J. Loaec N. Meijer L. Fruit C. Besson T. (2016). Synthesis of thiazolo[5,4-f]quinazolin-9(8H)-ones as multi-target directed ligands of Ser/Thr kinases. *Molecules* 21:578. 10.3390/molecules21050578 27144552 PMC6273584

[B24] Heeren J. Munzberg H. (2013). Novel aspects of brown adipose tissue biology. *Endocrinol. Metab. Clin. N. Am.* 42 89–107. 10.1016/j.ecl.2012.11.004 23391242 PMC3568264

[B25] Hess K. L. Wilson T. E. Sauder C. L. Gao Z. Ray C. A. Monahan K. D. (2009). Aging affects the cardiovascular responses to cold stress in humans. *J. Appl. Physiol.* 107 1076–1082. 10.1152/japplphysiol.00605.2009 19679742 PMC2763834

[B26] Horn H. Schoof E. M. Kim J. Robin X. Miller M. L. Diella F. (2014). KinomeXplorer: an integrated platform for kinome biology studies. *Nat. Methods* 11 603–604. 10.1038/nmeth.2968 24874572

[B27] Ino Y. Arakawa N. Ishiguro H. Uemura H. Kubota Y. Hirano H. (2016). Phosphoproteome analysis demonstrates the potential role of THRAP3 phosphorylation in androgen-independent prostate cancer cell growth. *Proteomics* 16 1069–1078. 10.1002/pmic.201500365 26841317

[B28] Inoue Y. Nakao M. Araki T. Ueda H. (1992). Thermoregulatory responses of young and older men to cold exposure. *Eur. J. Appl. Physiol. Occup. Physiol.* 65 492–498. 10.1007/BF00602354 1483436

[B29] Jain P. Karthikeyan C. Moorthy N. S. Waiker D. K. Jain A. K. Trivedi P. (2014). Human CDC2-like kinase 1 (CLK1): a novel target for Alzheimer’s disease. *Curr. Drug Targets* 15 539–550. 10.2174/1389450115666140226112321 24568585

[B30] Jiang K. Patel N. A. Watson J. E. Apostolatos H. Kleiman E. Hanson O. (2009). Akt2 regulation of Cdc2-like kinases (Clk/Sty), serine/arginine-rich (SR) protein phosphorylation, and insulin-induced alternative splicing of PKCβII messenger ribonucleic acid. *Endocrinology* 150 2087–2097. 10.1210/en.2008-0818 19116344 PMC2671910

[B31] Kamenecka T. M. Busby S. A. Kumar N. Choi J. H. Banks A. S. Vidovic D. (2010). “Potent anti-diabetic actions of a novel non-agonist PPARgamma ligand that blocks Cdk5-mediated phosphorylation,” in *Probe Reports from the NIH Molecular Libraries Program*, (Bethesda, (MD): National Center for Biotechnology Information, (US)). 23762958

[B32] Katano-Toki A. Satoh T. Tomaru T. Yoshino S. Ishizuka T. Ishii S. (2013). THRAP3 interacts with HELZ2 and plays a novel role in adipocyte differentiation. *Mol. Endocrinol.* 27 769–780. 10.1210/me.2012-1332 23525231 PMC5416755

[B33] Khim K. W. Choi S. S. Jang H. J. Lee Y. H. Lee E. Hyun J. M. (2020). PPM1A controls diabetic gene programming through directly dephosphorylating PPARgamma at Ser273. *Cells* 9:343. 10.3390/cells9020343 32024237 PMC7072254

[B34] Krook A. Kawano Y. Song X. M. Efendic S. Roth R. A. Wallberg-Henriksson H. (1997). Improved glucose tolerance restores insulin-stimulated Akt kinase activity and glucose transport in skeletal muscle from diabetic Goto-Kakizaki rats. *Diabetes* 46 2110–2114. 10.2337/diab.46.12.2110 9392506

[B35] Lande-Diner L. Boyault C. Kim J. Y. Weitz C. J. (2013). A positive feedback loop links circadian clock factor CLOCK-BMAL1 to the basic transcriptional machinery. *Proc. Natl. Acad. Sci. U.S.A.* 110 16021–16026. 10.1073/pnas.1305980110 24043798 PMC3791755

[B36] Lee K. M. Hsu la W. Tarn W. Y. (2010). TRAP150 activates pre-mRNA splicing and promotes nuclear mRNA degradation. *Nucleic Acids Res.* 38 3340–3350. 10.1093/nar/gkq017 20123736 PMC2879504

[B37] Li P. Carter G. Romero J. Gower K. M. Watson J. Patel N. A. (2013). Clk/STY (cdc2-like kinase 1) and Akt regulate alternative splicing and adipogenesis in 3T3-L1 pre-adipocytes. *PLoS One* 8:e53268. 10.1371/journal.pone.0053268 23308182 PMC3537621

[B38] Li P. Fan W. Xu J. Lu M. Yamamoto H. Auwerx J. (2011). Adipocyte NCoR knockout decreases PPARgamma phosphorylation and enhances PPARgamma activity and insulin sensitivity. *Cell* 147 815–826. 10.1016/j.cell.2011.09.050 22078880 PMC3783197

[B39] Lin J. Cao C. Tao C. Ye R. Dong M. Zheng Q. (2017). Cold adaptation in pigs depends on UCP3 in beige adipocytes. *J. Mol. Cell Biol.* 9 364–375. 10.1093/jmcb/mjx018 28486585

[B40] Liu Y. Conaway L. Rutherford Bethard J. Al-Ayoubi A. M. Thompson Bradley A. Zheng H. (2013). Phosphorylation of the alternative mRNA splicing factor 45 (SPF45) by Clk1 regulates its splice site utilization, cell migration and invasion. *Nucleic Acids Res.* 41 4949–4962. 10.1093/nar/gkt170 23519612 PMC3643583

[B41] Ma J. Chen T. Wu S. Yang C. Bai M. Shu K. (2019). iProX: an integrated proteome resource. *Nucleic Acids Res.* 47 D1211–D1217. 10.1093/nar/gky869 30252093 PMC6323926

[B42] Manning G. Whyte D. B. Martinez R. Hunter T. Sudarsanam S. (2002). The protein kinase complement of the human genome. *Science* 298 1912–1934. 10.1126/science.1075762 12471243

[B43] Manou-Stathopoulou V. Goodwin C. D. Patterson T. Redwood S. R. Marber M. S. Williams R. P. (2015). The effects of cold and exercise on the cardiovascular system. *Heart* 101 808–820. 10.1136/heartjnl-2014-306276 25673528

[B44] Marcheva B. Perelis M. Weidemann B. J. Taguchi A. Lin H. Omura C. (2020). A role for alternative splicing in circadian control of exocytosis and glucose homeostasis. *Genes Dev.* 34 1089–1105. 10.1101/gad.338178.120 32616519 PMC7397853

[B45] Menegay H. J. Myers M. P. Moeslein F. M. Landreth G. E. (2000). Biochemical characterization and localization of the dual specificity kinase CLK1. *J. Cell Sci.* 113(Pt 18) 3241–3253. 10954422 10.1242/jcs.113.18.3241

[B46] Mermoud J. E. Cohen P. T. Lamond A. I. (1994). Regulation of mammalian spliceosome assembly by a protein phosphorylation mechanism. *EMBO J.* 13 5679–5688. 7988565 10.1002/j.1460-2075.1994.tb06906.x PMC395533

[B47] Moeslein F. M. Myers M. P. Landreth G. E. (1999). The CLK family kinases, CLK1 and CLK2, phosphorylate and activate the tyrosine phosphatase, PTP-1B. *J. Biol. Chem.* 274 26697–26704. 10.1074/jbc.274.38.26697 10480872

[B48] Mulligan J. D. Gonzalez A. A. Stewart A. M. Carey H. V. Saupe K. W. (2007). Upregulation of AMPK during cold exposure occurs via distinct mechanisms in brown and white adipose tissue of the mouse. *J. Physiol.* 580 677–684. 10.1113/jphysiol.2007.128652 17272339 PMC2075554

[B49] Muraki M. Ohkawara B. Hosoya T. Onogi H. Koizumi J. Koizumi T. (2004). Manipulation of alternative splicing by a newly developed inhibitor of Clks. *J. Biol. Chem.* 279 24246–24254. 10.1074/jbc.M314298200 15010457

[B50] Murar M. Dobias J. Sramel P. Addova G. Hanquet G. Bohac A. (2017). Novel CLK1 inhibitors based on N-aryloxazol-2-amine skeleton – a possible way to dual VEGFR2 TK/CLK ligands. *Eur. J. Med. Chem.* 126 754–761. 10.1016/j.ejmech.2016.11.003 27940419

[B51] Nayler O. Stamm S. Ullrich A. (1997). Characterization and comparison of four serine- and arginine-rich (SR) protein kinases. *Biochem. J.* 326 693–700. 10.1042/bj3260693 9307018 PMC1218723

[B52] Nedergaard J. Bengtsson T. Cannon B. (2007). Unexpected evidence for active brown adipose tissue in adult humans. *Am. J. Physiol. Endocrinol. Metab.* 293 E444–E452. 10.1152/ajpendo.00691.2006 17473055

[B53] Nedergaard J. Bengtsson T. Cannon B. (2010). Three years with adult human brown adipose tissue. *Ann. N. Y. Acad. Sci.* 1212 E20–E36. 10.1111/j.1749-6632.2010.05905.x 21375707

[B54] Ohno H. Shinoda K. Spiegelman B. M. Kajimura S. (2012). PPARgamma agonists induce a white-to-brown fat conversion through stabilization of PRDM16 protein. *Cell Metab.* 15 395–404. 10.1016/j.cmet.2012.01.019 22405074 PMC3410936

[B55] Postolache T. Gautier S. Laloux B. Safar M. Benetos A. (1993). Positive correlation between the blood pressure and heart rate response to the cold pressor test and the environmental temperature in older hypertensives. *Am. J. Hypertens.* 6 376–381. 10.1093/ajh/6.5.376 8512662

[B56] Quaresma P. G. Weissmann L. Zanotto T. M. Santos A. C. de Matos A. H. Furigo I. C. (2017). Cdc2-like kinase 2 in the hypothalamus is necessary to maintain energy homeostasis. *Int. J. Obes.* 41 268–278. 10.1038/ijo.2016.174 27733761

[B57] Riachi M. Himms-Hagen J. Harper M. E. (2004). Percent relative cumulative frequency analysis in indirect calorimetry: application to studies of transgenic mice. *Can. J. Physiol. Pharmacol.* 82 1075–1083. 10.1139/y04-117 15644949

[B58] Rigbolt K. T. Prokhorova T. A. Akimov V. Henningsen J. Johansen P. T. Kratchmarova I. (2011). System-wide temporal characterization of the proteome and phosphoproteome of human embryonic stem cell differentiation. *Sci. Signal.* 4:rs3. 10.1126/scisignal.2001570 21406692

[B59] Robidoux J. Martin T. L. Collins S. (2004). Beta-adrenergic receptors and regulation of energy expenditure: a family affair. *Annu. Rev. Pharmacol. Toxicol.* 44 297–323. 10.1146/annurev.pharmtox.44.101802.121659 14744248

[B60] Roskoski R. (2015). A historical overview of protein kinases and their targeted small molecule inhibitors. *Pharmacol. Res.* 100 1–23. 10.1016/j.phrs.2015.07.010 26207888

[B61] Sako Y. Ninomiya K. Okuno Y. Toyomoto M. Nishida A. Koike Y. (2017). Development of an orally available inhibitor of CLK1 for skipping a mutated dystrophin exon in Duchenne muscular dystrophy. *Sci. Rep.* 7:46126. 10.1038/srep46126 28555643 PMC5448077

[B62] Saldivia M. Wollman A. J. M. Carnielli J. B. T. Jones N. G. Leake M. C. Bower-Lepts C. (2021). A CLK1-KKT2 signaling pathway regulating kinetochore assembly in *Trypanosoma brucei*. *mBio* 12:e0068721. 10.1128/mBio.00687-21 34128702 PMC8262961

[B63] Seale P. Conroe H. M. Estall J. Kajimura S. Frontini A. Ishibashi J. (2011). Prdm16 determines the thermogenic program of subcutaneous white adipose tissue in mice. *J. Clin. Invest.* 121 96–105. 10.1172/JCI44271 21123942 PMC3007155

[B64] Silvani A. Calandra-Buonaura G. Dampney R. A. Cortelli P. (2016). Brain-heart interactions: physiology and clinical implications. *Philos. Trans. A Math. Phys. Eng. Sci.* 374:20150181. 10.1098/rsta.2015.0181 27044998

[B65] Sono T. Akiyama H. Miura S. Deng J. M. Shukunami C. Hiraki Y. (2018). THRAP3 interacts with and inhibits the transcriptional activity of SOX9 during chondrogenesis. *J. Bone Miner. Metab.* 36 410–419. 10.1007/s00774-017-0855-2 28770354

[B66] Sun K. Kusminski C. M. Scherer P. E. (2011). Adipose tissue remodeling and obesity. *J. Clin. Invest.* 121 2094–2101. 10.1172/JCI45887 21633177 PMC3104761

[B67] Swoap S. J. Li C. Wess J. Parsons A. D. Williams T. D. Overton J. M. (2008). Vagal tone dominates autonomic control of mouse heart rate at thermoneutrality. *Am. J. Physiol. Heart Circ. Physiol.* 294 H1581–H1588. 10.1152/ajpheart.01000.2007 18245567

[B68] Tam B. Y. Chiu K. Chung H. Bossard C. Nguyen J. D. Creger E. (2020). The CLK inhibitor SM08502 induces anti-tumor activity and reduces Wnt pathway gene expression in gastrointestinal cancer models. *Cancer Lett.* 473 186–197. 10.1016/j.canlet.2019.09.009 31560935

[B69] Vohhodina J. Barros E. M. Savage A. L. Liberante F. G. Manti L. Bankhead P. (2017). The RNA processing factors THRAP3 and BCLAF1 promote the DNA damage response through selective mRNA splicing and nuclear export. *Nucleic Acids Res.* 45 12816–12833. 10.1093/nar/gkx1046 29112714 PMC5728405

[B70] Wang G. X. Zhao X. Y. Meng Z. X. Kern M. Dietrich A. Chen Z. (2014). The brown fat-enriched secreted factor Nrg4 preserves metabolic homeostasis through attenuation of hepatic lipogenesis. *Nat. Med.* 20 1436–1443. 10.1038/nm.3713 25401691 PMC4257907

[B71] Wong R. Balachandran A. Mao A. Y. Dobson W. Gray-Owen S. Cochrane A. (2011). Differential effect of CLK SR kinases on HIV-1 gene expression: potential novel targets for therapy. *Retrovirology* 8:47. 10.1186/1742-4690-8-47 21682887 PMC3148977

[B72] Xiong W. Zhao X. Garcia-Barrio M. T. Zhang J. Lin J. Chen Y. E. (2017). MitoNEET in perivascular adipose tissue blunts atherosclerosis under mild cold condition in mice. *Front. Physiol.* 8:1032. 10.3389/fphys.2017.01032 29311966 PMC5742148

