# Supplementary material for: Inhibition of a Novel CLK1-THRAP3-PPARγ Axis Improves Insulin Sensitivity
Source: Front Physiol. 2021 Aug 30;12:699578. doi: 10.3389/fphys.2021.699578 (PMC8435799; doi:10.3389/fphys.2021.699578)
Supplement: Supplementary file 3 [file Data_Sheet_3.PDF]

Supplemental Document

SUPPLEMENTAL MATERIALS AND METHODS

1. Animal study

To investigate the effects of mild cold temperature (16°C) on body weight gain and phosphoproteomics in adipose tissue, 8-week old male C57BL/6J mice (stock number 000664, from The Jackson Laboratory) were single housed in ventilated cages at either 22°C or 16°C, with a 12h/12h light/dark cycle, with the dark phase starting at 6pm, and with *ad libitum* access to standard chow diet (D12450J, Research Diets, 20% proteins, 10% fat, 70% carbohydrate) or high fat diet (HFD) (D12492, Research Diets, 20% proteins, 60% fat, 20% carbohydrate) and tap water. The ∆body weight, that is the change in body weight of each mouse (Figure 1A, chow diet; Figure 2A, high fat diet), was calculated by subtracting the body weight at the beginning of the experiment from that of each week. At the end of the experiment, the interscapular brown adipose tissue from mice fed a standard chow diet were collected for phosphoproteomics analysis (Figure 1B).

We generated *Clk1* knockout (CLK1 KO) mice (C57BL/6J background) with CRISPR/Cas9 technology. The guide RNA (gRNA) targeting the mouse *Clk1* gene was designed using the online software CRISPOR (http://crispor.tefor.net/crispor.py). The target sequence was GCATTGTGTTTGAACTTCTG, and is located in the coding exon 6 of the *Clk1* gene. Following the standard protocol developed in the lab[1], a mixture of sgRNA and *sp*Cas9 protein (purchased from Integrated DNA Technologies, idtdna.com) were injected into fertilized mouse eggs to produce CLK1 KO founders. The genotyping of the KO mice was performed by genomic PCR with the following primers: forward, 5'-GAAGCTGCTCAATCGGAAATAC-3'; and reverse, 5'-TCATGGGGACAGAACAAAAAG-3'. The PCR products were sequenced with the primer, 5'-AGAGTCCCTAGAGTCCCACCAGATTC-3' to identify the indel mutations at the targeting site. One of the founders had a 16bp insertion (CTTTGTAAGTATCACA) containing an in frame stop codon, TAA, which causes early termination of CLK1 translation. This founder was selected for further breeding to establish the CLK1 KO mouse line. To investigate the effects of CLK1 on body weight gain, insulin resistance and whole-body energy expenditure, the male CLK1 KO mice were fed a HFD for 8 weeks, starting at 8 weeks of age. The ∆body weight of each mouse (Figure 3A) was calculated as described above. At the end of HFD feeding period, whole-body energy expenditure was determined as described below, and oral glucose tolerance test (GTT) and insulin tolerance (ITT) test were performed as we previously reported[2] and detailed in the Materials and Methods section in the main doc. Tissues were collected upon sacrifice for histology and mRNA and protein determinations.

To investigate the effects of CLK1 inhibition on whole-body metabolism and insulin sensitivity, diet-induced obese (DIO) male C57BL/6J mice (stock number 380050) were purchased from the Jackson Laboratory. The DIO mice were fed an HFD for 24 weeks starting at 6-weeks of age. At week 24 of HFD feeding, the whole-body energy expenditure was measured as described below for 24 hours. Next, the mice were subcutaneously injected with the CLK1

inhibitor TG003 (50μg/kg in DMSO) [3] or DMSO once, and the whole-body energy expenditure was measured for an additional 24 hours. Then the mice were taken out from the chamber and continued to be treated with TG003 (subcutaneous injection, 50μg/kg in DMSO) or DMSO once a day for an additional 4 weeks while kept on HFD for determination of Δbody weight, GTT, ITT before sacrifice and expression of browning markers in adipose tissues collected at the time of sacrifice.

The animal numbers used for each experiment are indicated in the corresponding figure legends. The study protocol was approved by the Institutional Animal Care and Use Committee of the University of Michigan.

2. Energy expenditure assay in mice

Oxygen consumption (VO_2), carbon dioxide production (VCO_2), spontaneous motor activity and food intake were measured using the Comprehensive Laboratory Monitoring System (CLAMS, Columbus Instruments), an integrated open-circuit calorimeter equipped with an optical beam activity monitoring device as we described before[2]. Briefly, mice were individually housed into sealed chambers, and the environmental temperature was adjusted according to the experimental design, with free access to food and water. The study was conducted in an experimentation room with 12-12 hours (6:00PM~6:00AM) dark-light cycles. The measurements of energy expenditure were performed for 48 hours. The amount of food consumed by each animal was monitored through a precision balance attached below the chamber. Total energy expenditure was calculated based on the values of VO_2, VCO_2, and the protein breakdown[4]. The body composition was measured with a magnetic resonance imaging technique (EchoMRI 130, Echo Medical Systems).

3. Isolation and differentiation of preadipocytes

Preadipocytes isolated from the interscapular BAT of late fetal and newborn C57BL/6J mice and immortalized by introducing the SV40 antigen were kindly provided by Prof. Jiandie Lin at the University of Michigan [5]. Preadipocytes were grown on collagen-coated plates in DMEM containing 10% FBS and 1% Pen/Strep (Gibco) in a normoxic humidified cell culture incubator (7% CO_2 and 37°C). After reaching confluence, adipogenic differentiation was induced by supplementing the medium with IBMX (500μM), dexamethasone (1μM), insulin (20nM), T3 (1nM) and indomethacin (125μM). After 48 hours, the medium was replaced with fresh maintenance medium containing insulin and T3, which was replaced every other day. At day 5 of differentiation, the siRNA transfection (100nM siRNA pools) was performed using Dharmacon transfection reagents as per manufacturer's instructions. After 24 hours, at day 6, siRNA-mediated knockdown was repeated and 24 hours later, we performed treatment with 10μM CL316,243, a β3-adrenergic receptor agonist, for 24 hours followed by RNA extraction according to RNAeasy kit manual and analysis of mRNA expression of browning markers by quantitative Real-time PCR.

4. Western blot

Western blot analyses were performed as previously described[6]. Adipose tissue samples and

in vitro differentiated adipocytes were homogenized in lysis buffer (50mM Tris-HCl pH 7.4, 150mM NaCl, 2mM EDTA, 1.0% Triton X100, 0.5% sodium deoxycholate) supplemented with protease (Complete, Roche) and phosphatase (Halt phosphatase inhibitor cocktail, ThermoFisher) inhibitor cocktails. Lysates were cleared by centrifugation at 12,000g for 15 minutes at 4°C. Equal amount of proteins (30μg/lane) were separated on a 12% SDS-polyacrylamide gel, transferred to a nitrocellulose membrane (Bio-Rad) and stained for UCP1 (SAB3501101, 1μg/ml, Sigma-Aldrich), phospho-HSL (Ser660, #4126; 1μg/ml, Cell Signaling), total HSL (#4107, 1μg/ml, Cell Signaling), phospho-PPARγ (Ser273, LS-C209422-50; 2μg/ml, Lifespan Biosciences), total PPARγ (sc-7273, 1μg/ml, Santa Cruz), phospho-AKT (Ser473, #4060; 1μg/ml, Cell Signaling), total AKT (#4691, 1μg/ml, Cell Signaling), CLK1 (LS-C164346-400, Lifespan Biosciences), CLK2 (LS-C164347-400, Lifespan Biosciences), CLK3 (#3256, Cell Signaling), CLK4 (LS-C164349-400, Lifespan Biosciences), THRAP3 (A9396, ABclonal), HA-tag (#3724, Cell Signaling), Flag-tag (sc-166355, Santa Cruz) and β-tubulin (#2148, 0.1μg/ml, Cell Signaling). Signal from the HRP-conjugated secondary antibodies (0.1μg/ml, Calbiochem) was visualized by the Image Quant system (Bio-Rad).

5. Quantitative Real-time PCR

Total RNA from mice adipose tissue was extracted with Trizol reagent (Cat# 15596, Life Technologies, Grand Island, NY) and the first-strand cDNA was synthesized with the SuperScript® III First-Strand Synthesis System (Cat# 18080, Life Technologies, Grand Island, NY). Next, the relative abundance of multiple mRNAs in adipose tissue, as indicated in the corresponding figures, was analyzed by QRT-PCR using the primers listed in the supplemental table 3 and normalized by 18S rRNA, as the housekeeping gene. The relative expression levels of mRNAs were calculated using $2^{-\Delta\Delta CT}$ method.

6. Phosphoproteomics analysis

6.1. Sample preparation, High pH RP fractionation, TiO2 phosphopeptide enrichment

Brown adipose tissue was lysed in SDT buffer (4%SDS, 100mM Tris-HCl, 0.1M DTT, pH7.6) and homogenized using a Precellys24 Homogenizer (Bertin Technologies). After 3min of incubation at 95°C and sonication 20s per time for 3 times, the protein extracts were cleared by centrifugation for 10min at 12,000g. The protein concentration was determined by a fluorescent method[7]. Peptide digestion was performed by a filter-aided sample preparation protocol (FASP) with little modification[8]. For compatibility with tandem mass tags 10 (TMT10), protein digestion was performed in 200mM tetramethylammonium borohydride (TEAB). 1:50 (enzyme:protein) trypsin was added in two batches at 12h intervals. After 16-20h digestion, the peptides were dried and re-suspended in 20μl 500mM TEAB buffer and 400μg of digested peptides per sample were labeled by two sets of isobaric mass tags (TMT10, Thermo Fisher Sci). Two sets of TMT10-plex reagents were dissolved in 80μl anhydrous acetonitrile and added to the peptides making a final acetonitrile concentration of approximately 80% (v/v). After 1.5h incubation at room temperature with oscillation every 20 minutes, 16μl 5% hydroxylamine was used for 15min to quench the reaction. Equal concentration of labeled samples was mixed and dried. A 3.5mg TMT10 labeled peptide mixture was purified by 3M C18 StageTips (3M Empore) and fractionated by high-pH RP as

previously described with slight modifications[9]. The high-pH RP fractionation was performed with Dionex U3000 HPLC (Dionex, now ThermoFisher Scientific) using a Waters xbridge BEH300 C18 5μm 250*4.6mm column. The buffers used here were buffer A (2%ACN, adjusted to pH 10 by 2M $NH3{\cdot}H_2O$) and buffer B (98% ACN, adjusted to pH 10 by 2M $NH3{\cdot}H_2O$). The fractionation gradient was set as follows: 5% to 25% B for 40min, 25% to 40% B for 22min, 40% to 95% B for 2min, and 95% B for 4min, finally equilibrated in 5% B for 4min at a flow rate of 0.7 ml/min. The samples were collected every 2min, in up to 36 fractions. The collected fractions were merged as follows: F1-F13-F25, F2-F14-F26, F3-F15-F27, …, F12-F24-F36. Phosphopeptides in the 12 fractions were enriched by TiO2 as previously described[10]. The dried peptides were re-suspended in 500μl loading buffer (saturated glutamic acid / 65% ACN / 2% TFA). TiO2 beads (GL Sciences, Tokyo, Japan) were incubated in loading buffer at a final concentration of 100 μg/μl, of which 800μg were added to the fractions and allowed to react for 20min at room temperature. After the first enrichment by TiO2, the outflow of 12 fractions was recombined into 6 fractions as follows: F1-F7, ..., F6-F12. The second enrichment was performed by adding 1mg TiO2 beads. In addition, 0.5mg TMT10 of an unclassified peptide mixture was consecutively enriched by TiO2. In total, there were 20 samples of TMT10 labeled phosphorylated peptides to be subjected to mass spectrometry analysis.

6.2. Mass Spectrometry for Phosphoproteome

Phosphorylated peptides were separated by EASY-nLC 1000 C18 liquid chromatography (Thermo Fisher Scientific) and analyzed by Orbitrap Fusion (Thermo Fisher Scientific). The nanoscale C18 reverse phase column (75μm*150mm) was packed in-house with ReproSil-Pur C18-AQ 3.0 μm resin (Dr. Maisch GmbH, Germany). A linear gradient from 5% to 32% buffer B (buffer A, 0.1% formic acid in H2O and buffer B, 0.1% formic acid in acetonitrile) over 120min for fractionated samples and 180min for unfractionated samples were used for chromatography at a flow rate of 250nl/min. MS data was acquired with full scans (300-1500 m/z, R= 120,000 at 200 m/z) and 50ms maximum injection time at an AGC target of 2e5 ions. The most abundant parent ions were sequentially isolated with a window of 1.2 m/z and fragmented by higher energy collisional dissociation (HCD) with 33% NCE. A resolution of 60,000 at 200 m/z and 100ms maximum injection time at an AGC target of 1e5 ions was used for MS/MS scans.

6.3. Data Processing and Bioinformatics

For phosphoproteome analysis, raw mass spectrometry data were processed using the MaxQuant software version 1.5.2.8 and peak lists were analyzed against the mouse Uniprot database (version August 2016). Methionine oxidation and N-terminal protein acetylation and serine/ threonine/tyrosine (STY) phosphorylation were set as variable modifications and Cysteine carbamidomethylation as fixed modifications. Peptides with at most two missed cleavages of trypsin were considered for identification. The false discovery rate was 1% for all proteins, peptides and modification sites against a reversed sequence database. The quality tolerance for peptide first search of 20ppm, quality tolerance for peptide main search of 6ppm and the reporter ion tolerance of 0.002Da were set as search parameters. Using a commonly strict criterion (localization probability > 0.75, score diff > 5), 6810 class I sites without missing

value in all samples were applied to the downstream bioinformatics analyses in R (version 3.5.1). The levels of phosphorylation sites were corrected by the average of the total intensity of samples. Phosphorylation was considered as “regulated by cold” in chow diet-fed mice if the fold change was more than 1.5 and the p value of Student’s t-test corrected by Benjamini-Hochberg (BH) method was less than 0.05. Kinase-substrate relationships were predicted using NetworKIN 3.0 with NetworKIN scores > 1 and max difference < 4. Hypergeometric test was performed to calculate the significance of kinase enrichment and the p value was further corrected by Benjamini-Hochberg (BH) method. The adjusted p value was less than 0.05.

7. Mass Spectrometry for Identification of CLK1 Interacting Partners

HEK293T cells were transfected with pcDNA3-HA-CLK1 or empty vector as control using Lipofectamine 2000 (Invitrogen) according to manufacturer’s instructions. Cell lysates were harvested in lysis buffer (Cell lysis buffer for Western and IP, P0013, Beyotime) and were immunoprecipitated with anti-HA monoclonal antibody-conjugated agarose (Abmart) 48h after the transfection and prepared to identify endogenous interacting proteins of CLK1 by LC-MS/MS. The pulled down proteins were then processed by a filter-aided sample preparation (FASP) protocol and purified by 2M C18 Stage Tips. The nLC system EASY-nLC 1000 C18 liquid chromatography (Thermo Fisher Scientific) was coupled to an Orbitrap Fusion mass spectrometer (Thermo Fisher Scientific) with the following gradient for 40min at a rate of 300 nl/min: 5% B (0-2min), 5-30% B (2-27min), 30-45% B (27-30min), 45-90% B (30-32min) and equilibrated in 90% B for 8min (buffer A: 0.1% FA in H2O, buffer B: 0.1% FA in ACN). Other parameter settings and search software were as described above, except for the following modifications: resolution 120,000 at 200 m/z, scan range 300-1500 m/z, maximum injection time 50ms, AGC target 2e5 for full scan and resolution 15,000 at 200 m/z, isolation window 1.2 m/z, maximum injection time 80ms, AGC target 1e5 for MS/MS scan in data-dependent acquisition mode.

8. Mass Spectrometry for Identification and Quantification of S243 in THRAP3

HEK293T cells were transfected with pcDNA3-HA-THRAP3 and pCDH-flag-CLK1 as experimental groups or pcDNA3-HA-THRAP3 and pCDH empty vectors as control using Lipofectamine 2000 (Invitrogen) according to manufacturer’s instructions. Cell lysates were harvested in lysis buffer (Cell lysis buffer for Western and IP, P0013, Beyotime) and were immunoprecipitated by anti-HA monoclonal antibody-conjugated agarose (Abmart) 24h after transfection. Two biological replicates were analyzed in two or three technical replicates. The protein was digested using the filter-aided sample preparation protocol (FASP) and purified by 3M C18 StageTips. The samples were separated by EASY-nLC 1000 C18 liquid chromatography (Thermo Fisher Scientific) and analyzed by Q Exactive HF-X (Thermo Fisher Scientific). The peptides were eluted with the following gradient for 45min at a flow rate of 300 nl/min: 2-5% B (0-2min), 5-35% B (2-39min), 35-90% B (39-41min) and equilibrated in 90% B for 4min (buffer A: 0.1% FA in H2O, buffer B: 0.1% FA in ACN). For MS data, 400 to 1200 m/z full scan was acquired with a resolution of 120,000 at 200 m/z, a maximum injection time of 50ms and an AGC target of 3e6. For MS/MS data, data-independent acquisition (DIA) mode was applied by 40 consecutive isolation windows of 20 m/z width with a resolution of 15,000 at 200 m/z and 30% NCE. For the library of DIA, mass spectrometry was set as previously

mentioned. Skyline 3.7.0 was performed to analyze DIA mass spectrometry data using the following search parameters: trypsin digestion with at most two miss-cleavages, a time window of 2min, admitted peptide length from 7 to 45 AAs for peptide setting, and precursor charges of 2,3,4, ion charges of 1,2,3, ion types of y,p,b, a ion match tolerance of 0.02 m/z for transition settings. The top six product ions from filtered ion charges/types and its parent ions were used to quantify the phosphorylation of S243 in THRAP3. All detected peaks were reintegrated using the mProphet algorithm and the p-value was less than 0.05 for peptide and modification identification.

SUPPLEMENTAL FIGURE LEGENDS

Supplemental Figure 1

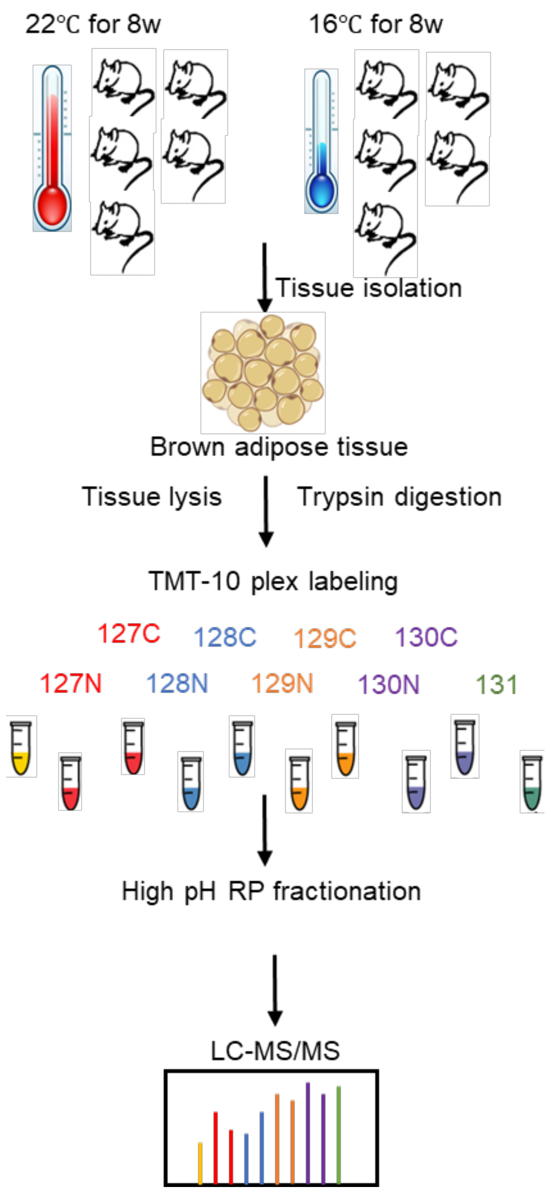

Supplemental Figure 1. Strategy for the phosphoproteomics.

Supplemental Figure 2

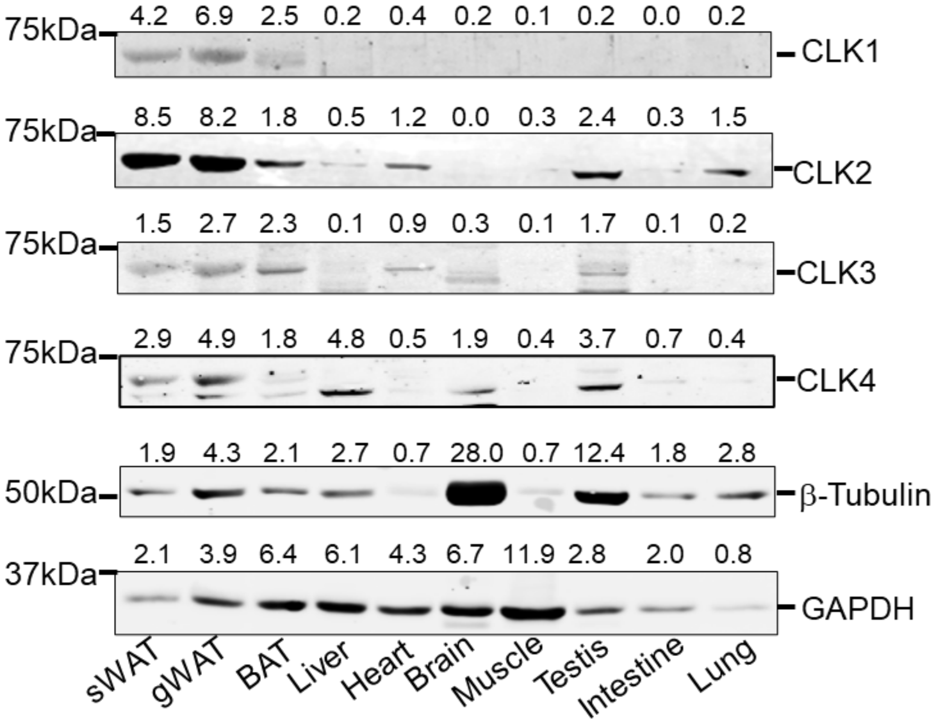

Supplemental Figure 2. The protein levels of the CLK family of kinases in sWAT, gWAT, BAT, liver, heart, brain, lung, skeletal muscle, and intestine of 8-week-old C57BL/6J mice. β-Tubulin and GAPDH are shown as controls. The numbers above the lanes indicate the densitometry values (Image Studio, Li-Cor).

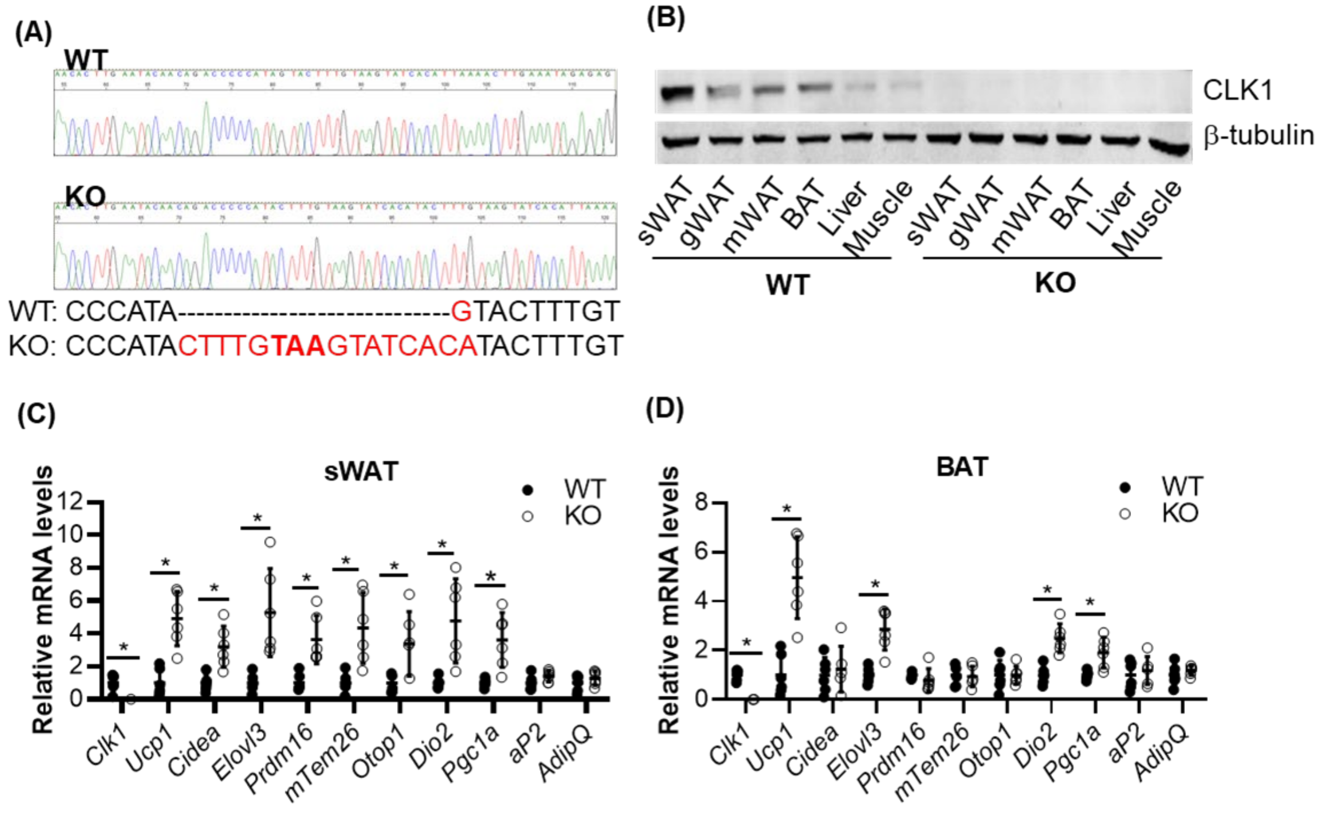

Supplemental Figure 3. *Clk1* knockout induces adipose tissue browning. **(A)** Generation of *Clk1* knockout mice by CRISPR/Cas9. Chromatogram traces of the CLK1 wild-type and knockout homozygous by Sanger sequencing. The gRNA sequence, targeting exon 6, is indicated by the red lettering. The alignment between mouse *Clk1* wild-type and knockout sequences is shown under the chromatograms. There is a 16bp insertion in the knockout mouse, which includes an in-frame stop codon, TAA (bolded). **(B)** Western blot showing absence of CLK1 protein in sWAT, gWAT, mWAT (mesenteric WAT), BAT, liver and skeletal muscle in *Clk*1 knockout mice. **(C-D)** The mRNA levels of browning markers (relative to 18S rRNA) in sWAT (C) and BAT (D) of 8-week-old wild-type and *Clk1* knockout mice fed a standard chow diet at 22°C. Data shown as mean±SD, *p<0.05. n=5 mice/group. (Unpaired Student's *t* test for each gene)

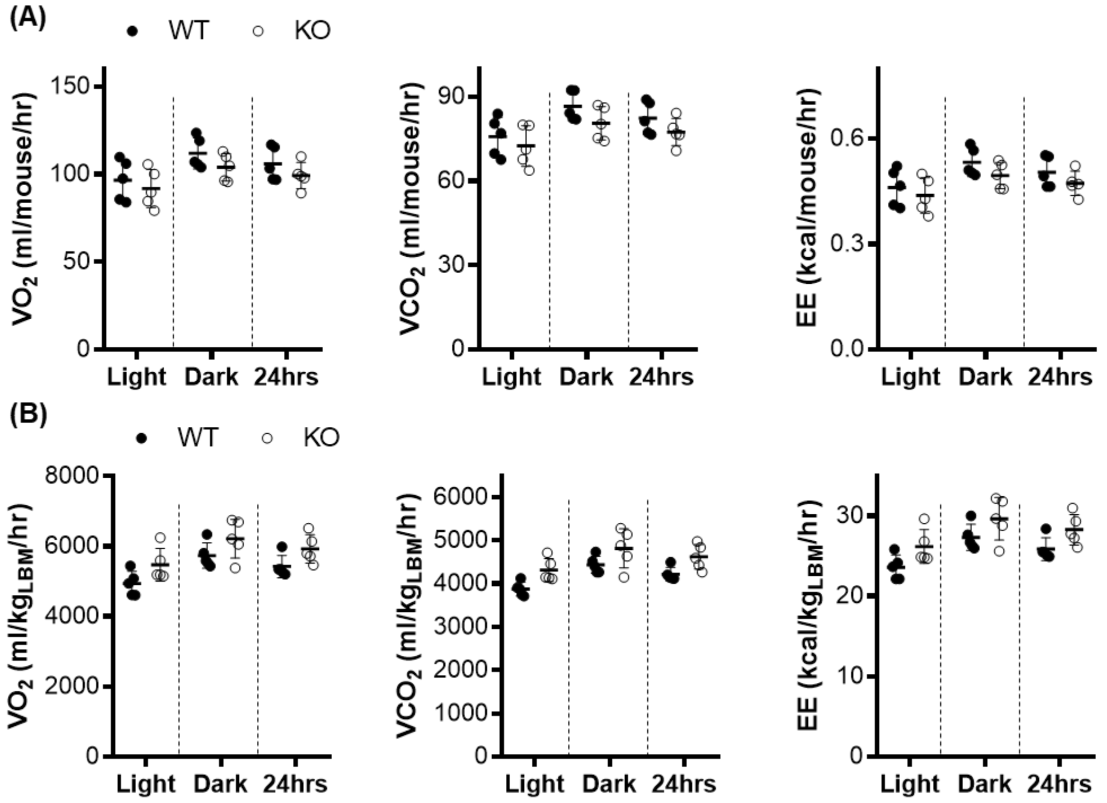

Supplemental Figure 4. The whole-body energy expenditure of CLK1 KO mice. The male CLK1 KO (KO) and wild-type (WT) mice were fed a high-fat diet (HFD) for 8 weeks, starting at 8 weeks of age. The whole-body energy expenditure of mice was measured at the end of the 8 weeks' HFD feeding. The environmental temperature in the metabolic chambers was set at 22°C. The metabolic index was monitored for 24 hours, and the results were averaged during the light-on period, or during the light-off period and for the 24h period. n=5 mice/group. Data shown as mean±SEM. **p*<0.05 *vs* WT. Data are shown as absolute values without normalization to body weight **(A)** or normalized by lean body mass **(B)**. No statistical significance was found between groups (One-way ANOVA with Sidak's multiple comparisons test).

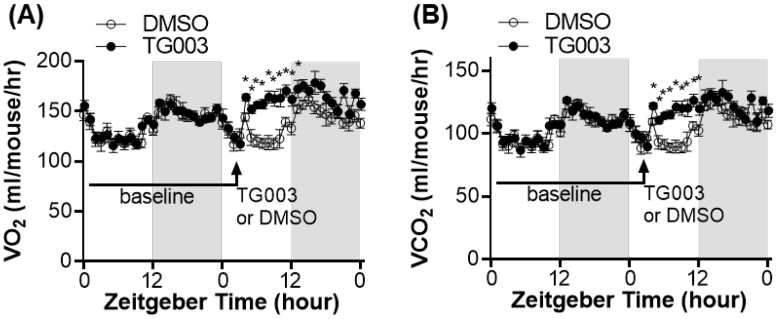

Supplemental Figure 5. The whole-body energy expenditure of mice upon CLK1 inhibitor treatment. Diet induced obesity (DIO) in male C57BL/6J mice was induced by high-fat diet (HFD) for 24 weeks starting at 6 weeks of age. Energy expenditure in obese mice was measured at baseline for 24 hours after 24 weeks of HFD feeding. After subcutaneous injection of one bolus of the CLK inhibitor TG003 (50μg/kg) or vehicle control DMSO (black arrow), the energy expenditure was measured for another 24 hours. Gray area indicates light-off period. n=5 mice/group. Data shown as mean±SEM. Data are shown as absolute values without normalization to body weight. *$p<0.05$ *vs* DMSO (One-way ANOVA with Sidak's multiple comparisons test).

Supplemental Table 3. PCR primers used in Figure 1D, Figure 2G, Figure 8 and Supplemental Figure 3 C and D.

Primers	Forward primer	Reverse primer
AdipoQ	TGTTCCTCTTAATCCTGCCCA	CCAACCTGCACAAGTTCCCTT
Ap2	TCACCTGGAAGACAGCTCCT	AAGCCCACTCCCACTTCTTT
Cidea	CTGTCGCCAAGGTCGGGTCAAG	CGAAAAGGGCGAGCTGGATGTAT
Cited	ACCTTGGAGTGAAGGATCGC	GAGGGGTAGGATGCAGGTTG
Clk1	TCCAGAGGAGATGGCAACCTA	GCGCTCTGATGATGTAGTCTG
Clk2	CAGCAGTTACCGAAGCCAGC	CAGCGTCGTCCTCTACACTCTT
Clk3	CACCACTGTCACAAACGCCGTAC	CCACGATCTCATATCGCTCTTGG
Clk4	AGCGGCTGGAGAGGAACG	CAATGGATCCGGTAAGTGCTTTC
Cox8b	TGGGGATCTCAGCCATAGTC	CTCAGGGATGTGCAACTTCA
Dio2	ATGTAACCAGCACCGGAAAG	ATGCAGAAAGGCAGACTCGT
Elovl3	GGGCCTCAAGCAAACCGTGTG	GTTTGGCAGCCTTCATAGTGTAGT
Otop1	TTTCTACCGGATGCACGCAG	ACAGTGTTTGCTCATCCCCG
Pgc1a	AGCCTCTTTGCCCAGATCTT	GGCAATCCGTCTTCATCCAC
Prdm16	CCGGCCTTCTACTCACAGCATTCC	GCGGGTTTTTCCTCTTTTGGTT
Tbp	TGTTCTCCTGCCTTCCCTATC	CTGAGTGTCCTCACCAACTGT
Tmem26	GCTCACCCTCAAGTTCAAGC	GGGTTCTGCAATACTGGTTT
Ucp1	AAAAACAGAAGGATTGCCGAAACT	TAAGCATTGTAGGTCCCCGTGTAG

REFERENCES

[1] D. Yang, J. Xu, T. Zhu, J. Fan, L. Lai, J. Zhang, *et al*, Effective gene targeting in rabbits using RNA-guided Cas9 nucleases. J Mol Cell Biol 6 (2014) 97-9. DOI: 10.1093/jmcb/mjt047

[2] W. Xiong, X. Zhao, M.T. Garcia-Barrio, J. Zhang, J. Lin, Y.E. Chen, *et al*, MitoNEET in Perivascular Adipose Tissue Blunts Atherosclerosis under Mild Cold Condition in Mice. Front Physiol 8 (2017) 1032. DOI: 10.3389/fphys.2017.01032.

[3] M. Murar, J. Dobias, P. Sramel, G. Addova, G. Hanquet, and A. Bohac, Novel CLK1 inhibitors based on N-aryloxazol-2-amine skeleton - A possible way to dual VEGFR2 TK/CLK ligands. Eur J Med Chem 126 (2017) 754-761. DOI: 10.1016/j.ejmech.2016.11.003.

[4] M. Riachi, J. Himms-Hagen, and M.E. Harper, Percent relative cumulative frequency analysis in indirect calorimetry: application to studies of transgenic mice. Canadian journal of physiology and pharmacology 82 (2004) 1075-83. DOI: 10.1139/y04-117.

[5] G.X. Wang, X.Y. Zhao, Z.X. Meng, M. Kern, A. Dietrich, Z. Chen, *et al*, The brown fat-enriched secreted factor Nrg4 preserves metabolic homeostasis through attenuation of hepatic lipogenesis. Nature medicine 20 (2014) 1436-1443. DOI: 10.1038/nm.3713.

[6] L. Chang, L. Villacorta, R. Li, M. Hamblin, W. Xu, C. Dou, *et al*, Loss of perivascular adipose tissue on peroxisome proliferator-activated receptor-gamma deletion in smooth muscle cells impairs intravascular thermoregulation and enhances atherosclerosis. Circulation 126 (2012) 1067-78. DOI: 10.1161/CIRCULATIONAHA.112.104489

[7] S.S. Thakur, T. Geiger, B. Chatterjee, P. Bandilla, F. Frohlich, J. Cox, *et al*, Deep and Highly Sensitive Proteome Coverage by LC-MS/MS Without Prefractionation. Mol Cell Proteomics 16(7) (2011) 1-9. DOI: 10.1074/mcp.M110.003699.

[8] J.R. Wisniewski, A. Zougman, N. Nagaraj, and M. Mann, Universal sample preparation method for proteome analysis. Nature methods 6 (2009) 359-62. DOI: 10.1038/nmeth.1322.

[9] T.S. Batth, C. Francavilla, and J.V. Olsen, Off-line high-pH reversed-phase fractionation for in-depth phosphoproteomics. J Proteome Res 13 (2014) 6176-86. DOI: 10.1021/pr500893m.

[10] Q.R. Li, Z.B. Ning, J.S. Tang, S. Nie, and R. Zeng, Effect of peptide-to-TiO2 beads ratio on phosphopeptide enrichment selectivity. J Proteome Res 8 (2009) 5375-81. DOI: 10.1021/pr900659n.
